# A review of microcavitation bubbles dynamics in biological systems and their mechanical applications

**DOI:** 10.1016/j.ultsonch.2025.107521

**Published:** 2025-08-21

**Authors:** Ahmed K. Abu-Nab, Adel M. Morad, Ehab S. Selima, Tetsuya Kanagawa, Ali F. Abu-Bakr

**Affiliations:** a Moscow Center for Advanced Studies, Kulakova str. 20, Moscow 123592, Russia; b Department of Mathematics and Computer Science, Faculty of Science, Menoufia University, Shebin El-Koom 32511, Egypt; c Department of Engineering Mechanics and Energy, Faculty of Engineering, Information and Systems, University of Tsukuba, 1-1-1 Tennodai, Tsukuba 305-8573, Japan; d Menoufia National University, Menoufia, 6162101, Egypt

**Keywords:** Microcavitation bubbles, Histotripsy, Lipid-coated multi microbubbles, Encapsulated microbubbles, Divers’ biotissue, Viscoelastic tissue

## Abstract

In this review, the theoretical studies are presented for the microcavitation bubble dynamics problems, which mainly depend on the microcavitation models, such as the Rayleigh-Plesset model, the Church model, the diffusion-concentration model, and the Keller-Miksis model in biological systems. The various solutions to these models, which were formulated based on basic mathematical and physical concepts, are schematically presented. Moreover, these models are employed in many different physical problems, such as the treatment of cancerous tumours via a technique known as histotripsy and lipid shells of membrane cells, that employ focused ultrasonic therapy as a non-invasive tissue ablation method. Using the mechanical action of bubble clouds, historic triumph destroys tissue differently than the thermally ablative techniques of therapeutic ultrasonography. Also, lipid-coated microbubbles are used in different therapeutic applications. Besides, the effect of different physical coefficients on Newtonian, non-Newtonian fluids, and viscoelastic media studied by numerical and analytical methods with external fields is examined for incompressible fluid states, and the Navier-Stokes hydrodynamic equations are investigated. It is anticipated that this work will serve as a valuable guide for the use of microcavitations in many medicinal applications, such as histotripsy, lipid shells in membrane cells and diver’s tissue.

## Nomenclature

$B_{2}$: Constant defined in Eq. (12) (–)
C: Sound speed $\left(m/s\right)$
$C_{B}$: Instantaneous concentration of gas around microbubble, defined in Eq. (5) (–)
$C_{c}$: Concentration distribution (–)
$a_{l}$: Thermal diffusivity $\left(m^{2}/s\right)$
E: Effective viscoelastic stress (–)
G: Young modulus $\left(kPa\right)$
H: Distance between tow bubbles number (m)
N: Number of bubbles (–)
$k_{s}$: Shell viscosity $\left(N/m^{2}\right)$
u: Velocity of mixture (m/s)
$g_{g}$: Gravity ($m/s^{2}$)
r: Radial coordinate (m)
R: Bubble radius (m)
$\dot{R}$: Bubble velocity $\left(m/s\right)$
$\ddot{R}$: Bubble acceleration $\left(m/s^{2}\right)$
Q: Constant, defined in Eq. (5) (–)
t: Time $\left(s\right)$
$Z_{i},i=1,2,3,4,5,6$: Constants defined in Eqs. (15),16) (–)

**Greek symbols**
k: Thermal conductivity, (W/mk)
α: Stiffening parameter (–)
σ: Surface tension $\left(m^{3}/s\right)$
ρ: Density ($\mathrm{Kg}/m^{3}$)
$\lambda_{i},i=1,2,3,4$: Constants defined in Eq. (6) (–)
η: Viscosity in fluid $\left(N/m^{2}\right)$
$J_{a}$: Jacob number (–)
$\Phi_{0}$: Initial void fraction (–)
χ: Lipid monolayer elasticity (–)
P: Pressure (Pa)
ε: Ratio of density in liquid, defined in Eq. (30) (–)

**Subscripts**
g: Gas
m: Maximum value
0: Initial value

**Abbreviations**
RPE: Rayleigh-Plesset equation
LCS: lipid-coated shells
KME: Keller-Miksis equation
NH: Neo-Hookean
QLKV: quadratic law Kelvin-Voigt

## 1 Introduction

Many natural phenomena and different applications in our daily lives are interacting particles of kinetic and hydrodynamic flows [[1], [2], [3], [4], [5], [6], [7]]. Studies of the dynamics of these particles in hydrodynamic flows under the influence of different fields will enhance our understanding of many of these natural phenomena, such as climate change, global warming, coastal erosion, and other natural phenomena surrounding us. Moreover, these studies will help improve production in the industrial, agricultural, and various other sectors. Currently, environmental change caused by human activities, for example, is receiving a great deal of interest in the scientific community. Building theoretical models to interpret and predict physical and experimental data is a primary goal in many branches of science. However, these models are only useful if they are based on reliable and physically valid theoretical concepts [[8], [9], [10], [11]]. The processes of advanced oxidation were studied in cavitation bubble dynamics with the revelation of ultrasonic behaviour [12]. The investigation into the theory of bubble dynamics [[13], [14], [15], [16], [17], [18], [19], [20], [21], [22], [23]] has become among the most relevant topics in current scientific and technological domains, due to its wide applications across various areas, including biological sciences, mechanical and environmental engineering, industrial and chemical technologies, and marine-related engineering fields [[23], [24], [25], [26], [27], [28]].

Microcavitation and bubble dynamics have been considered important topics in physics and engineering for more than a century. Since the end of the nineteenth century, corrosion problems in ship propellers led to the theoretical and practical study of strong ultrasonic fields in fluids after World War I [[29], [30], [31], [32]]. As a result, studies and research developed significantly, which helped improve technological equipment, such as modern computers and fast imaging devices [33].

For the oscillation of a spherical gas bubble in an infinite body of fluid, the most widely used nonlinear equations of motion originate from the Rayleigh-Plesset equation (RPE), which is defined in terms of the bubble radius's dependence on the conditions present in the gas and liquid. In recent decades, a number of important analyses and research projects have been built upon a heuristically derived version of the RPE, whose parameter of interest is the bubble volume rather than the radius and which does not derive the dissipation term from first concepts. To characterize the nonlinear response of a gas bubble in a liquid to a driving pressure field, the RPE is thus the nonlinear equation that is most frequently used.

(1) $$\rho_{l}\left(R\ddot{R}+\frac{3}{2}{\dot{R}}^{2}\right)=P_{g}-P_{\infty }-\frac{2}{R}\sigma$$

here R is the bubble radius, dot refer to the derivatives. $P_{g}$ is the gas pressure. $P_{\infty }$ is ambient pressure. $\rho_{l}$ is the density of liquid. σ is the surface tension. In contrast, some computational formulations based on an RPE version use the bubble volume as the dynamic variable rather than recalculating the damping [32,[34], [35], [36], [37]]. On the other hand, the Keller-Miksis equation (KME) provides an equation for the enormous, radial oscillations of a bubble enclosed in a sound field. When the frequency of the sound field approaches the intrinsic frequency of the bubble, large amplitude oscillations will happen. The KME, which was not previously included in the RPE computations earlier, considers the surface tension, acoustic radiation originating from the bubble, viscosity, and incident sound waves [38,39].

The majority of the microcavitation research that has been done thus far has focused determining how pressure amplitude change affects the microcavitation resonance frequency and associated bubble size, as well as how to use it in therapeutic systems. Numerous biological systems and medicinal applications experience microcavitation [[40], [41], [42]]. We point out that while bubble cavitations can be usefully applied in a variety of medical domains when properly harnessed, inertial cavitation can result in significant mortality or harm to cells and soft tissues. One of the most significant therapeutic uses of microcavitation is in surgical methods for conditions like neurological disorders, fat-coated shells in viscoelastic tissue, cardiovascular disorders, lithotripsy and histotripsy.

Ultrasonography is also being used in medicine for more and more diagnostic as well as therapeutic purposes. Sonography, among the most popular uses are shock waves for lithotripsy, cell ablation, ultrasound-assisted medication delivery, wound healing, and tissue regeneration [43,44]. It is commonly known that bubble formation and collapse near a tumor cell border are indicative of a re-entrant jet, the direction of which is determined by the type of boundary and the strength of the standoff distance. It was highlighted that the conditions treated in the histotripsy clinical trials included hepatocellular carcinoma, benign prostatic hyperplasia, and calcified aortic stenosis. Histotripsy creates cavitation bubbles by applying ultra-rapid, high-pressure ultrasonic pulses, which are typically shaped erratically due to nonlinear propagation effects. This is done using techniques that are distinct from traditional therapeutic ultrasound [[45], [46], [47]]. Unlike ultrasonic thermal therapy, theoretical analysis indicates a potential mechanism for tumor cell liquefaction induced by histotripsy that looks like a cellular homogenate.

As a result, new approaches are required for creating exposure protocols for histotripsy and managing its treatment. However, non-spherical behavior which is the focus of this study can be anticipated because of the interactions between microbubbles. Furthermore, the development of numerical models for ultrasonic contrast agents has been the focus of numerous investigations. Often, they are restricted to spherical models that describe the behavior of a single bubble [38]. Numerous efforts have been made to create techniques for solving KME, and RPE in a reasonable manner for a variety of physical issues [40,42,48].

The aim of this review is to know the main concepts of cavitation bubble dynamics and their mechanical applications in the fields of biological and medical systems. We present some physico-mathematical models and theoretical results describing the various dynamics processes which are described by the theory of bubble dynamics in mechanical and biological applications.

## 2 Formulation microcavitation bubble dynamics

The formation of microscopic pockets or bubbles in a liquid due to a drop in pressure is known as microcavitation. When pressure builds up, these bubbles explode, unleashing a large “local” force that can cause a variety of natural phenomena, such as electron gasification, acoustic luminescence, and the damage of biotissue. Microbubbles can grow and contain gas, vapour, or a combination of the two by statically or dynamically decreasing the surrounding pressure. When dissolved gases from the liquid diffuse into the bubble, temperature rises, pressure drops, or other factors can cause the gas in the bubbles to expand. However, if most of the bubbles are composed of vapour, then raising the temperature to a point where the vapour bubbles keep expanding will result in cavitation, a phenomenon where evaporation into the cavities happens when the ambient pressure is sufficiently reduced at a nearly constant temperature. This ongoing phenomenon is known as boiling. This suggests that until the lower limit is reached, neither boiling nor evaporation occur. Several techniques can be used to trigger the formation of microbubbles. For instance, in the event of a gas-filled bubble, either by increasing temperature or decreasing pressure, this is referred to as gaseous cavitation. And, by lowering pressure in the case of a vapour-filled bubble, this is known as vaporous cavitation. But, when a bubble is filled with gas, by diffusion. The process known as degassing occurs when gas escapes the liquid. And, when a bubble full of vapour reaches a certain temperature, boiling happens. The situation is very complicated because microbubbles usually contain a mixture of gas and vapor. Looking at it another way, we can distinguish between four different types of microcavitation depending on how they are generated. First, pressure variations in the moving fluid due to the system's shape generate hydrodynamic cavitations [[49], [50], [51], [52]]. The movement of sound waves through the fluid because of pressure changes is known as acoustic cavitation [53,54]. The optical cavity happens when photons with a high optical density, or a laser, break through the liquid. Furthermore, another kind of elementary particle, such as a proton, cavitates the particles and bursts the liquid, much like in a bubble chamber.

A critical process in two-phase flows is the exchange of mass and heat between gases and liquids. For a variety of reasons, numerous researchers have examined the development of microcavitation in superheated/supersaturated liquids. The growth rate of microvariations is dependent on a few factors, including surface tension, liquid inertia, and the difference in pressure between the bubble's interior and exterior. Due to the intricacy of the phenomenon, simplified heat transfer models have been used to conduct mathematical evaluations for the growth/collapse of a single bubble for simple geometric shapes. Some of the main theories regarding the inertial or thermal/mass diffusion related growth of gas bubbles resulting from various pertinent and physical challenges are presented in this paper.

In Rayleigh's first analysis of a cavitation and bubble dynamics problem, the equation of motion of a spherical microbubble was found to be the momentum interaction between the bubble and the surrounding liquid [[55], [56], [57]]. Heat transfer through the bubble interface and surface tension were not considered. Besides, based on the motion of the bubble radius and the presumption that there is no heat diffusion over the bubble surface, we found two theoretical solutions for the rate of expansion of these unstable bubbles [28,58]. And experimental data on the formation of vapour bubbles in superheated water were compared with these two solutions. This comparison, which considers the heat dispersion effect, demonstrates agreement with the obtained solutions. Additionally, Forster and Zuber [59] solved the integral differential equation for the vapor microbubble's development in a superheated liquid. For various high temperatures, there was good agreement with experimental results in the case of negligible hydrodynamic forces.

The work of researchers [40] achieved an important breakthrough when they solved the Rayleigh equation by taking into account the heat transfer via the bubble interface in a liquid that had been uniformly warmed. Heat conduction across the thermal boundary layer around the bubble provided the equation for the creation of bubbles. Using the notion of a uniformly superheated bulk liquid and the thin thermal boundary layer assumption, they calculated the temperature gradient at the interface. A formula for the radius of a vapour bubble as a function of the square root of time was also derived by them; this formula is applicable to sufficiently large radii and agrees well with experimental results in liquids that have been overheated.

Additionally, most microbubble dynamics, cavitation techniques, and the correlations between theoretical and experimental outcomes were thoroughly examined. For thermal and inertial pitches, they calculated the bubble growth rate [59,60]. In addition, a mathematical framework for the development of bubbles in biological tissues was presented by Srinivasan et al. model [61]. Because the two dynamic models that are suitable for these applications assume that the bubble is either inside an immobile tissue (two-zone model) or enclosed by a boundary layer inside a well-moving tissue (three-zone model), they have two relationships for the rate of change in the bubble radius. Moreover, they showed that while the two-zone model is only relevant for bubble evolution in tissues with infinite extent, the three-zone model is a better fit for bubble evolution in restricted tissue volumes.

Conversely, the Plesset and Zwick theory was adjusted by solving the momentum equation between two finite boundaries and adding an initial value for the bubble's expanded velocity. And used Scriven's similarity parameter method modification to solve the heat diffusion equation [62]. Furthermore, presented was the temperature distribution of the superheated liquid between two-phase temperatures at two finite boundaries as a spherical vapour bubble grew. In these specific conditions, the temperature distribution and the evolution of the vapor bubble are obtained through an analytical approach, where the resulting expressions take an implicit form, which is different from the ones previously reported. A more general formula has been applied for estimating the bubble growth rate, and its predictions are compared with the results presented in [43,62].

## 3 Microcavitation bubbles dynamics in biological system

Bubbles and cavities are present in a broad variety of biological media and soft materials [[63], [64], [65]]. Scientists are trying to understand and control the behaviour of these fascinating objects, which range from bubbles formed during the breakdown of volcanic eruptions' magma to those found in the synovial fluid that forms. In many industrial operations, where bubbles expand or contract complex fluids, controlling bubble dynamics is essential. The dispersion of carbon nanotubes during ultrasonication is influenced by cavitation and vapor bubble collapse, which leads to nanotube scission, while the production of polymer foams, for instance, is dependent on the growth of gas bubbles in a polymeric liquid. Ultrasound can be used to manage tiny bubbles injected into the body to deliver medication to normally inaccessible areas, such as across the blood–brain barrier. A thorough understanding of bubble dynamics near or within soft tissue is essential to the success of a surgery in this instance, as it is in many other medical applications of ultrasound. Additionally, cavitation has a role in biological processes that our understanding of is still developing.

Larger-scale cavitation bubbles can result in an embolism in the water circulation when trees and plants are subjected to severe hydric evaporative stress. Soft and biological materials are defined by both elasticity and viscosity because they behave in a way that is halfway between that of a fluid and a solid, such as cells, tissues, colloidal suspensions, polymer solutions, and gels. Such viscoelastic materials' mechanical characteristics usually also depend on the applied deformation's amplitude and pace.

The complicated response of these materials to flow and deformation, as well as the microscopic source of this response, are studied in rheology. Even in simple fluids, the highly nonlinear bubble dynamics can be dramatically altered by the viscoelastic material's nonlinear rheological activity. In biological environments, bubbles are commonplace. They can be purposefully created in bioreactors or arise from the intricate dynamics of waves breaking in open oceans. From formation, through motion, until death, bubbles play a critical role in the oxygenation and mixing of natural and artificial ecosystems. However, their life is also greatly influenced by the environments in which they emerge. The delicate relationship that exists between microorganisms and bubbles depends heavily on surface tension. In addition to determining how microorganisms impact each stage of the bubble life cycle, it also shapes the role that bubbles play in blending or oxygenating microorganisms. Ultrasound effects have shown a major potential extremely invasive bio-medical and biological therapy way, which can be utilized in different areas, i.e., tissue-cutting (namely histotripsy), thermal ablation, lithotripsy, and administering medication. The essential success and use of medical therapies are based on the scale size of cavitation bubbles: i.e., miller, micro and nanoscale. Cavitation bubbles dynamics of ultrasound effects introduce a better understanding of how medical therapy is a progressive method and provides feedback in a way. Cavitation bubbles dynamics is not an extremely, minimally, direct invasive therapeutic method. Moreover, the effect of mechanical characteristics of biological tissues on stress and strain wave and cavitation bubbles dynamics as following optical breakdown and, power and pulsed laser ablation [[66], [67], [68], [69]] is a significant topic where gets lately garnered attention.

This review aims to clarify and analyze the qualitative discrepancies observed among various mathematical models and theoretical investigations concerning the behavior of microcavitation bubbles. Specifically, it addresses the effects of both isolated bubble dynamics and interactions between multiple bubbles within biomedical and biological contexts, including applications such as histotripsy, lipid shells in cellular membranes, and tissues exposed during diving.

The assumption that the surrounding medium behaves as incompressible is supported by the very short timescale over which cavitation bubble dynamics occur, during which the tissue medium effectively exhibits incompressible characteristics. Regarding bubble shape, the near-spherical geometry is justified by the predominance of surface tension forces and the relatively minor deformations experienced during oscillations. This simplification facilitates mathematical modeling without compromising the essential physical accuracy. Furthermore, the assumption of scale separation as the characteristic size of the bubbles is considerably smaller than the wavelength of the ultrasound field, enabling the decoupling of micro-scale bubble dynamics from the larger-scale acoustic effects. Collectively, these assumptions provide a consistent physical framework while maintaining the analytical tractability of the model.

The key assumptions can be summarized as follows:

• The tissue medium is treated as nearly incompressible over the brief duration of cavitation events.
• Bubbles retain an approximately spherical shape due to dominant surface tension and limited deformation during oscillatory motion.
• The size of the bubbles is significantly smaller than the ultrasound wavelength, allowing for the separation of micro- and macro-scale phenomena.

## 4 Microcavitation dynamics during the histotripsy

**Fig. 1:**
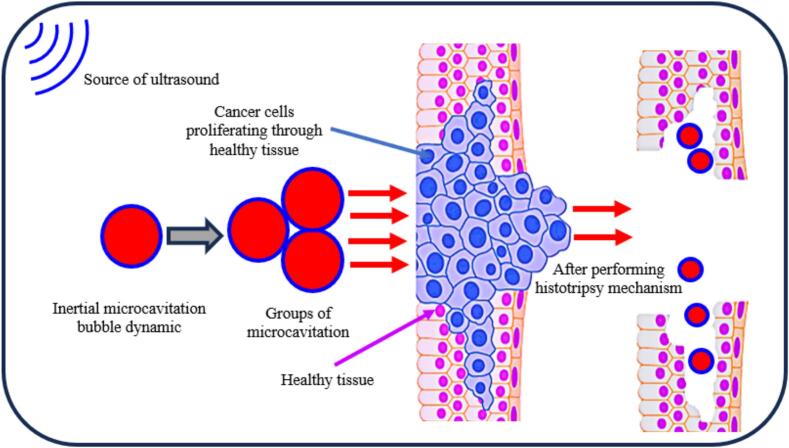
Mechanism of histotripsy via microcavitation bubble dynamics based on the high acoustic ultrasound effects.

To enhance non-invasive ultrasonic ablation methods such as histotripsy, which uses microcavitation to mechanically separate tissue into a cellular detritus, it is crucial to monitor bubble cavitation dynamics. With enough pulses, histotripsy can completely fractionate tissue into a homogenate that looks liquid and lacks cellular characteristics. In Fig. 1, we examine the cavitation bubble dynamics in a viscoelastic medium exposed to a histotripsy pulse using various fidelities to characterize compressibility and thermal effects.

The process of histotripsy refers to the non-thermal rising-intensity based ultrasonic/ultrasound therapy, where utilized in the objected induction of cavitation bubbles events to statically and dynamically fractionate and damage soft bio-tissues (for example in structure schematic Fig. 1). The theoretical description and mathematical approach of cavitation microbubbles were described by researchers in ref. [13,70,71] in viscoelastic tissue through the process of histotripsy. The influences of viscoelastic properties such as shear stress of viscosity, Young modulus, and stiffening effects were used in formulation cavitation dynamics during histotripsy. The primary objective of this study is to examine the effects of viscoelastic properties such as shear viscosity, Young’s modulus, and nonlinear stiffening—on the behavior of cavitation bubbles produced by histotripsy. The use of ultrasound-induced cavitation in biomedical applications involving nanomaterials has seen notable growth recently, especially in therapeutic approaches where soft tissues exposed to acoustic waves interact dynamically with nanoparticles. This interaction is important for understanding the mechanical effects on tissues and improving therapeutic outcomes. In order to describe the key mechanical responses of soft biological environments, two different constitutive models were adopted: the Neo-Hookean (NH) model and the Quadratic Law Kelvin-Voigt (QLKV) model. Here, we introduce two models.

• Neo-Hookean Model: This model is widely applied in computational biomechanics to describe soft tissues characterized by nearly incompressible behavior and moderate nonlinear elasticity. It is usually used to simulate brain tissue and other soft connective tissues. Because of its simple mathematical structure, it is appropriate for problems involving large deformations, assuming the tissue response is isotropic and hyperplastic. The relatively simple mathematical form of the NH model allows effective simulation of large deformation while still assuming isotropic and hyperplastic response, which is suitable for describing conditions near cavitation regions where local strains may be high.
• Quadratic Law Kelvin-Voigt (QLKV) Model: The QLKV model is aimed at tissues where viscoelastic effects are non-negligible, for example, arterial walls and soft tissues with significant internal damping. This formulation accounts for both elastic storage and viscous loss behavior and includes a nonlinear stiffness term to better match with biological observations under ultrasound loading. The time-dependent characteristics represented by this model are especially important under high-frequency pressure fluctuations from cavitation oscillations in histotripsy.

The mathematical framework of both models is based on a modified (KME), which describes radial bubble dynamics in compressible and viscoelastic environments. The incorporation of tissue rheology into KME allows for improved characterization of the cavitation field in nanomedicine settings, including nanoparticle delivery and controlled mechanical damage. This model serves as a necessary component for evaluating how nanomaterial interactions can be modulated by tissue mechanical responses under focused ultrasound. The modified Plesset-Zwick approach (PZA) is used to obtain analytical solutions for the governing model. The growth of cavitation microbubbles during the histotripsy procedure is inhibited by the proposed parameters of Young modulus, viscosity effects, and stiffening configurations according to an analysis of the results.

### 4.1 A non-interaction microcavitation bubble dynamics in the histotripsy

The proposed model of the cavitation microbubble during histotripsy is based on the influence of viscoelastic biotissue, which have viscosity $v_{L}$, Young modulus G, and stiffening effects α, and in parallel to behaviour of cavitation microbubbles [72]. Based KME of cavitation bubbles dynamics [73], the proposed model was formulated in NH model and QLKV model, in radial coordinates during histotripsy as

(2) $$\left(1-\frac{\dot{R}}{C}\right)R\ddot{R}+\frac{3}{2}\left(1-\frac{\dot{R}}{3C}\right){\dot{R}}^{2}=\frac{1}{\rho_{l}}\;\left(1+\frac{\dot{R}}{C}+\frac{R}{C}\frac{d}{\mathrm{dt}}\right)\left(P_{a}\left(t\right)-P_{\infty }\right)$$

here C is the constant of the sound speed, where it is undistributed around the used medium. $\rho_{l}$, $P_{\infty }$ are the density around viscoelastic medium and the saturated pressure respectively. R is the radius of cavitation bubble and dot referring to the derivatives with respect to time t. Here, it is found that the proposed models of cavitation dynamics during histotripsy depended on configuration of viscoelastic bio-tissues as viscosity parameter η, stiffening factor α and Young modulus G. The term of $\left(\dot{R}/C\right)$ refers to the Mach number. In the case of very low Mach number that means $\left(\dot{R}/C\right)$ approaches to zero. Then the Eq. (2) reduces to

(3) $$R\ddot{R}+\frac{3}{2}{\dot{R}}^{2}=\frac{1}{\rho_{l}}\;\left(1+\frac{R}{C}\frac{d}{\mathrm{dt}}\right)\left(P_{a}\left(t\right)-P_{\infty }\right)$$

The influential pressure on the cavitation microbubble dynamics $P_{a}\left(t\right)$ is defined as

(4) $$P_{a}\left(t\right)=P_{B}\left(t\right)-\frac{2}{R}\sigma +E-\frac{4\dot{R}}{R}\eta$$

where κ is to polytropic exponent. $R_{0}$ is the initial value of microbubble radius. The terms E and $\frac{4\eta \dot{R}}{R}$ refer to the combination of viscous and elastic contribution in this study. The term of effective viscoelastic stress E give us the case I of NH model and QLKV model which can be defined as

Case I: NH model [[72], [73], [74], [75], [76]], the term E of can be put

$$E=-\frac{1}{2}G\left(5-4\left(\frac{R_{0}}{R}\right)-4{\left(\frac{R_{0}}{R}\right)}^{4}\right)$$

Case II: QLKV model [[72], [73], [74], [75], [76]], the term E of can be put

$$E=\frac{1}{2}G\left(3\alpha -1\right)\left(5-4\left(\frac{R_{0}}{R}\right)-4{\left(\frac{R_{0}}{R}\right)}^{4}\right)+2G\alpha \left(\frac{27}{40}+\frac{1}{8}{\left(\frac{R_{0}}{R}\right)}^{8}+\frac{1}{5}{\left(\frac{R_{0}}{R}\right)}^{5}+{\left(\frac{R_{0}}{R}\right)}^{2}-\frac{2R}{R_{0}}\right)$$

here $\nu_{L}$ is the liquid viscosity. G refers to shear modulus. α is stiffening parameter. Additionally, the term E is named the nonlinear strain-stiffening QLKV model which relies on the different values of agarose gel concentrations. It is found that the physical parameters for QLKV and NH models are different. This is due to several factors in the nature of the problems studied, including, for example, the type of cancerous tumour present, the place where this cancerous tumour originates, and its size. All these previous factors are important in how and determining to choose the appropriate model for the study. Moreover, in this study, we have relied on the different physical values for both models, which have already been used in previous studies [[72], [73], [74], [75], [76], [77]]. The type of gel concentration plays the dominant role in the study of the NH model and QLKV model in cavitation bubbles dynamics that relied on the factors of viscoelastic tissue: for example, in Table 1 in ref. [76].

**Table 1:** Experimental values of parameters of agarose gel concentration [76].

Parameter	NH-model		QLKV-model	
	0.1 % Gel	0.3 % Gel	0.1 % Gel	0.3 % Gel
α	0	0	1.5	2.8
$G\left(kPa\right)$	9.1	31	0.44	7.5
$\eta \left(Pa\;s\right)$	0.077	0.15	0.079	0.15
$R_{0}\left(\mu m\right)$	0.25	1.3	0.21	1.3

To solve NH model of cavitation dynamics, the difference of pressure [78,79] is supposed as

(5) $$P_{B}\left(t\right)-P_{\infty }=bc^{n}+Q\left(C_{B}-C_{\infty }\right)$$

where b and n are the dominant sonic speed c exponent factors which were determined in ref. [9] as b=0.9 and n=2. Respectively. $C_{B}$ refers to the instantaneous concentration of gas around microbubble. Q is a constant, which will estimate based on initial and boundary conditions. The details of solution of Neo-Hookean model of cavitation bubbles are presented in ref. [48] where the resultant of the behaviour of cavitation bubble undertaking the configurations of NH model through histotripsy can be written

(6) $$R\left(t\right)=\lambda_{1}\left\{\lambda_{2}+\frac{1}{{\Gamma^{2}R_{0}^{2}\;\rho }_{L}}\;\left(\lambda_{3}\left(-\frac{2\sigma }{R_{0}}\beta_{m}^{-\frac{1}{3}}-4\left(C_{1}+\frac{C_{2}}{R_{0}^{2}}\beta_{m}^{-\frac{2}{3}}\right)-\frac{1}{2}G\left(5-4\beta_{m}^{-\frac{1}{3}}-4\beta_{m}^{-\frac{4}{3}}\right)\right)+\frac{{R_{0}\beta }_{m}^{\frac{1}{3}}}{c}\left(\lambda_{4}+8C_{2}\frac{\Gamma }{3}\beta_{m}^{\frac{1}{3}}\overset{´}{\beta_{m}}-\frac{2}{3}G\Gamma \overset{´}{\beta_{m}}\left(1+\frac{4}{\beta_{m}}\right)\right)\right)\right\}t^{\frac{1}{2}}$$

where, $\lambda_{1}=\frac{2\rho_{l}{R_{0}}^{3}{\left(\frac{\Gamma^{5}}{3}\right)}^{\frac{1}{2}}}{Q_{2}\pi \left(1+\frac{\Gamma R_{0}\beta_{m}^{\frac{2}{3}}{\overset{´}{\beta }}_{m}}{3c}\right)}$,

$$\lambda_{2}=\frac{R_{0}\beta_{m}{\overset{´}{\beta }}_{m}\Gamma }{9c}\left(\beta_{m}^{2}{\;\beta }_{m}^{"}+\frac{2}{3}\;\beta_{m}{\overset{´}{\;\beta }}_{m}^{2}\right)+\frac{1}{18c}\beta_{m}^{\frac{4}{3}}{\overset{´}{\beta }}_{m}^{2}+\frac{1}{\rho_{l}\Gamma^{2}R_{0}^{2}}\left(1+\frac{\Gamma R_{0}\beta_{m}^{\frac{2}{3}}{\overset{´}{\beta }}_{m}}{3c}\right)\left(bc^{n}+Q_{1}\right)$$

$\lambda_{3}=\left(1+\frac{\Gamma R_{0}\beta_{m}^{\frac{2}{3}}{\overset{´}{\beta }}_{m}}{3c}\right)$ and $\lambda_{4}=-\frac{\kappa P_{0}}{\rho_{l}c{\;R}_{0}\Gamma }\beta_{m}^{\frac{2}{3}\;-\kappa }{\overset{´}{\beta }}_{m}+\frac{2\sigma }{{3R}_{0}}\Gamma \overset{´}{\beta_{m}}$.

And also the radius of microcavitation bubble on histotripsy process under considering properties of QLKV model as

(7) $$R\left(t\right)=K_{1}\left[K_{2}+\frac{1}{\rho_{L}\Gamma^{2}R_{0}^{2}}\left(1+\frac{\Gamma R_{0}{\varphi_{0}}^{\frac{-2}{3}}{\overset{´}{\beta }}_{m}}{3c}\right)\left(B_{1}+bc^{n}\right)-K_{3}+\frac{G\left(3\alpha -1\right)}{2\rho_{L}\Gamma^{2}R_{0}^{2}}\left(5\left(1-\frac{\Gamma R_{0}}{3c}{\varphi_{0}}^{\frac{-2}{3}}{\overset{´}{\beta }}_{m}\right)-4{\varphi_{0}}^{\frac{1}{3}}-{\varphi_{0}}^{\frac{4}{3}}\left(1-\frac{\Gamma R_{0}}{c}{\varphi_{0}}^{\frac{-2}{3}}{\overset{´}{\beta }}_{m}\right)\right)+\frac{2G\alpha }{\rho_{L}}\left(\frac{27}{40}\left(1+\frac{\Gamma R_{0}}{3c}{\varphi_{0}}^{\frac{-2}{3}}{\overset{´}{\beta }}_{m}\right)+\frac{1}{8}{\varphi_{0}}^{\frac{8}{3}}\left(1-\frac{7\Gamma R_{0}}{3c}{\varphi_{0}}^{\frac{-2}{3}}{\overset{´}{\beta }}_{m}\right)+\frac{1}{5}{\varphi_{0}}^{\frac{5}{3}}\left(1-\frac{4\Gamma R_{0}}{3c}{\varphi_{0}}^{\frac{-2}{3}}{\overset{´}{\beta }}_{m}\right)+\beta_{m}^{\frac{-2}{3}}\left(1-\frac{\Gamma R_{0}}{3c}{\varphi_{0}}^{\frac{-2}{3}}{\overset{´}{\beta }}_{m}\right)-2{\varphi_{0}}^{\frac{-1}{3}}\left(1+\frac{2\Gamma R_{0}}{3c}{\varphi_{0}}^{\frac{-2}{3}}{\overset{´}{\beta }}_{m}\right)\right)\right]J_{a}\sqrt{\frac{24}{\pi }}\sqrt{a_{L}t}$$

where, the constants $K_{1}$, $K_{2}$, $K_{3}$ are illustrated in ref. [38].

**Fig. 2:**
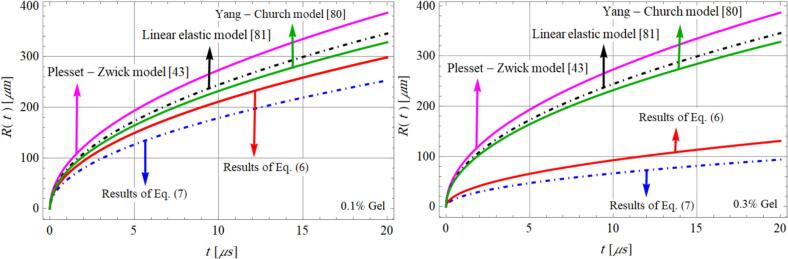
Comparison results for models in Eqs. (6), (7) and previous studies in [43,80,81] for two gel concentration, $J_{a}=15.14$, $a_{l}=1.397\times 10^{-7}m^{2}/s$. These results are calculated and published in refs. [38,72,74].

The main outcomes of both the NH and QLKV models are summarized in Fig. 2. These results demonstrate that the generalized solution for microcavitation in a viscoelastic medium under varying gel concentrations aligns well with previous theoretical predictions, including those reported by PZA [43], Yang [80], and models based on linear elasticity [81].

**Fig. 3:**
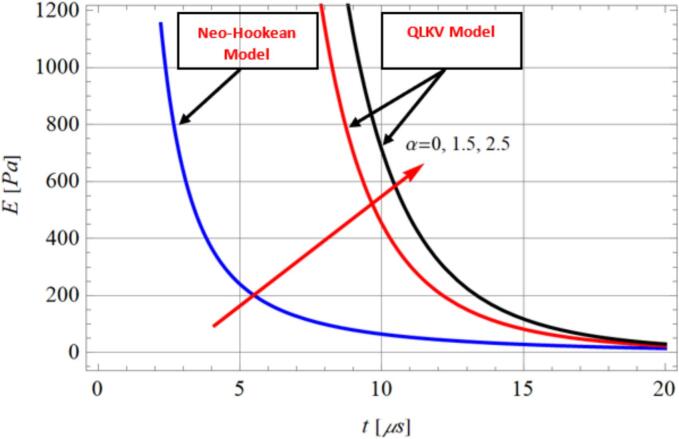
E vs time t, published in ref. [29] for values of stiffening factors α through cavitation microbubbles on histotripsy; $a_{l}=1.397\times 10^{-7}m^{2}/s$, $v_{L}=0.77Pa\;s$, $J_{a}=15.14$.

Fig. 3 and Table 2 further present the analytical results for the effective viscoelastic stress E as a function of time during histotripsy-induced cavitation. It was observed that effective viscoelastic stress E increases with both the stiffening factor α and the Young’s modulus in both the Neo-Hookean and QLKV models, indicating that material stiffening significantly affects the mechanical response of the medium [82].

**Table 2:** The proposed models and their solutions compared with previous studies of cavitation behaviour during histotripsy.

Model	E(t)	$C_{1}$
Linear elastic [81]	$-2G\left(1-\frac{R_{0}^{2}}{C_{1}^{2}t}\right)-2\frac{\eta }{t}$	$K_{1}J_{a}\sqrt{\frac{24a_{L}}{\pi }}\left[K_{2}+\frac{1}{\rho_{l}\Gamma^{2}R_{0}^{2}}\left(1+\frac{\Gamma R_{0}{\Phi_{0}}^{\frac{-2}{3}}{\overset{´}{\beta }}_{m}}{3C}\right)\left(A_{1}+bc^{n}\right)-K_{3}+\frac{4G{\left(1-\Phi_{0}\right)}^{\frac{2}{3}}}{3}\right]$
NH-model (6)	$-\frac{G}{2}\left(5-\frac{4{C_{1}}^{3}t^{3/2}R_{0}+R_{0}^{4}}{{C_{1}}^{4}t^{2}}\right)-2\frac{\nu_{L}}{t}$	$K_{1}\left[K_{2}+\frac{1}{\rho_{l}\Gamma^{2}R_{0}^{2}}\left(1+\frac{\Gamma R_{0}{\Phi_{0}}^{\frac{-2}{3}}{\overset{´}{\beta }}_{m}}{3C}\right)\left(A_{1}+bc^{n}\right)-K_{3}-\frac{G}{2\rho_{l}\Gamma^{2}R_{0}^{2}}\left(5\left(1-\frac{\Gamma R_{0}}{3C}{\Phi_{0}}^{\frac{-2}{3}}{\overset{´}{\beta }}_{m}\right)-4{\Phi_{0}}^{\frac{1}{3}}-{\Phi_{0}}^{\frac{4}{3}}\left(1-\frac{\Gamma R_{0}}{C}{\Phi_{0}}^{\frac{-2}{3}}{\overset{´}{\beta }}_{m}\right)\right)\right]J_{a}\sqrt{\frac{24}{\pi }a_{l}}$
QLKV model (7)	$\frac{1}{2}G\left(-1+3\alpha \right)\left(5-\frac{4{C_{1}}^{3}t^{3/2}R_{0}+R_{0}^{4}}{{C_{1}}^{4}t^{2}}\right)-2\frac{\nu_{L}}{t}+\frac{1}{20}G\alpha \left(27-\frac{80C_{1}\sqrt{t}}{R_{0}}+\frac{40{C_{1}}^{6}t^{3}R_{0}^{2}+8{C_{1}}^{3}t^{3/2}R_{0}^{5}+5R_{0}^{8}}{{C_{1}}^{8}t^{4}}\right)$	$K_{1}\left[K_{2}+\frac{1}{\rho_{l}\Gamma^{2}R_{0}^{2}}\left(1+\frac{\Gamma R_{0}{\Phi_{0}}^{\frac{-2}{3}}{\overset{´}{\beta }}_{m}}{3C}\right)\left(B_{1}+bc^{n}\right)-K_{3}+\frac{G\left(3\alpha -1\right)}{2\rho_{l}\Gamma^{2}R_{0}^{2}}\left(5\left(1-\frac{\Gamma R_{0}}{3C}{\Phi_{0}}^{\frac{-2}{3}}{\overset{´}{\beta }}_{m}\right)-4{\Phi_{0}}^{\frac{1}{3}}-{\Phi_{0}}^{\frac{4}{3}}\left(1-\frac{\Gamma R_{0}}{C}{\Phi_{0}}^{\frac{-2}{3}}{\overset{´}{\beta }}_{m}\right)\right)+\frac{2G\alpha }{\rho_{l}}\left(\frac{27}{40}\left(1+\frac{\Gamma R_{0}}{3C}{\Phi_{0}}^{\frac{-2}{3}}{\overset{´}{\beta }}_{m}\right)+\frac{1}{8}{\Phi_{0}}^{\frac{8}{3}}\left(1-\frac{7\Gamma R_{0}}{3C}{\Phi_{0}}^{\frac{-2}{3}}{\overset{´}{\beta }}_{m}\right)+\frac{1}{5}{\Phi_{0}}^{\frac{5}{3}}\left(1-\frac{4\Gamma R_{0}}{3C}{\Phi_{0}}^{\frac{-2}{3}}{\overset{´}{\beta }}_{m}\right)+\beta_{m}^{\frac{-2}{3}}\left(1-\frac{\Gamma R_{0}}{3C}{\Phi_{0}}^{\frac{-2}{3}}{\overset{´}{\beta }}_{m}\right)-2{\Phi_{0}}^{\frac{-1}{3}}\left(1+\frac{2\Gamma R_{0}}{3C}{\Phi_{0}}^{\frac{-2}{3}}{\overset{´}{\beta }}_{m}\right)\right)\right]J_{a}\sqrt{\frac{24}{\pi }}a_{l}$
Yang and Church [80]	$-\frac{4}{3}G\left(1-\frac{R_{0}^{3}}{C_{1}^{3}}t^{-\frac{3}{2}}\right)-2\frac{\nu_{L}}{t}$	$K_{1}J_{a}\sqrt{\frac{24a_{L}}{\pi }}\left[K_{2}+\frac{1}{\rho_{L}\Gamma^{2}R_{0}^{2}}\left(1+\frac{\Gamma R_{0}{\Phi_{0}}^{\frac{-2}{3}}{\overset{´}{\beta }}_{m}}{3C}\right)\left(A_{1}+bc^{n}\right)-\frac{4\nu_{L}}{{3\rho }_{L}R_{0}^{2}\Gamma }\left(\frac{\Gamma R_{0}}{C}\left({\Phi_{0}}^{-2}\beta_{m}^{"}+\frac{2}{3}{\Phi_{0}}^{-1}{\overset{´}{\beta }}_{m}^{2}\right)+{\Phi_{0}}^{\frac{-1}{3}}{\overset{´}{\beta }}_{m}\right)+\frac{4G\left(1-\Phi_{0}\right)}{3}\right]$

Importantly, the predictions of the QLKV model show strong agreement with experimental findings on cavitation bubble dynamics reported in [52,57]. Moreover, the estimated values of E in Table 2 for selected parameters G,η,α are consistent with the analytical forms reported in [80,81]. This comparison underscores the added value of incorporating viscoelasticity and nonlinear stiffening in modeling tissue response. While the Neo-Hookean model captures elastic behavior, it lacks time-dependent dissipation. The QLKV model addresses this limitation by accounting for viscous effects, making it more suitable for modeling realistic biological media. However, both models still assume isotropy and neglect poroelastic and microstructural heterogeneities, pointing to open areas for future model refinement.

€.

### 4.2 Interacting microcavitation bubble dynamics in the histotripsy

In this section, it is found that there are some researchers investigating the interacting of micro cavitation bubble dynamics during histotripsy as in ref. [47,83,84] who were introduced the analytical investigation of theoretical mathematical models for cavitation bubble clouds with effect of interparticle interaction during histotripsy. The theoretical models are established and discussed to non-interacting of single microbubbles and interacting microbubble clouds with using the impact of variety of surface tension as constants and variable cases of surface tension. The models of single and interacting cavitation bubbles were derived on the basis of KME through NH [66,74] and the QLKV [76,84], then these models are converted to ordinary differential equations by applying the non-dimensions variables methodology, where they are solved analytically by applying the modified PZA.

The mechanism of the cavitation bubble clouds dynamics in the scale of nano or micro scale during histotripsy where it found that the mechanism of histotripsy can break down tissue into a liquid-appearing homogenate with no existing cellular features with enough pulses. Authors [14] supposed the influence of viscoelastic tissue of the spring G,α and a dashpot η is considered in this work. The proposed model of single cavitation of microbubbles dynamics during histotripsy with impact of stress viscoelastic tissue can be expressed in the modified KME [2] in no existing interaction gas-filled cavitation microbubbles as

(8) $$\left(1-\frac{\dot{R}}{C}\right)R\ddot{R}+\frac{3}{2}\left(1-\frac{\dot{R}}{3C}\right){\dot{R}}^{2}=\frac{1}{\rho_{l}}\;\left(1+\frac{\dot{R}}{C}+\frac{R}{c}\frac{d}{\mathrm{dt}}\right)\left(P_{B}-P_{\infty }-2\frac{\sigma \left(t\right)}{R}+\frac{1}{2}G\left(3\alpha -1\right)\left(5-4\left(\frac{R_{0}}{R}\right)-\;\;4{\left(\frac{R_{0}}{R}\right)}^{4}\right)+2G\alpha \left(\frac{27}{40}+\frac{1}{8}{\left(\frac{R_{0}}{R}\right)}^{8}+\frac{1}{5}{\left(\frac{R_{0}}{R}\right)}^{5}+{\left(\frac{R_{0}}{R}\right)}^{2}-\frac{2R}{R_{0}}\right)-4\eta \frac{\dot{R}}{R}\right)$$

here the pressure in the region around the microbubbles [38] was given as

(9) $$P_{B}\left(t\right)=P_{g0}{\left(\frac{R_{0}}{R}\right)}^{3\kappa }$$

where κ is to polytropic exponent. $R_{0}$ is the initial value of microbubble radius. $P_{g0}$ is an ambient pressure of gas. To find the pressure due to the interaction of cavitation bubbles during histotripsy, firstly the Euler equation and mass equation (namely the continuity equation) can be expressed in radial-spherical coordinates for incompressible fluids [14], and then we get the velocity of the mixture in radial coordinates as

$$u=\frac{1}{r^{2}}\left(R^{2}\frac{\mathrm{dR}}{\mathrm{dt}}\right)$$

Moreover, we can get the pressure because of interparticle interaction for the interaction between i- and j-microbubbles as

(10) $$P_{proposed}=\sum_{J=1,J\neq i}^{2}P_{j}=\frac{\rho_{l}}{r_{j}}\sum_{J=1,J\neq i}^{N}\frac{d}{\mathrm{dt}}\left(R_{j}^{2}\left(\frac{dR_{j}}{\mathrm{dt}}\right)\right)$$

Through this work [14], authors supposed that the interaction Fig. 1 and Table 2 illustrate the analytical results of effective viscoelastic stress E in term time on cavitation bubbles during histotripsy. It was observed that the stress of effective viscoelastic increases at increasing the effect of stiffening factor α and Young modulus in Neo-Hookean model and QLKV model of cavitation dynamics. The given results agree with the published experimental results [76,83] on cavitation bubbles behaviour. QLKV model estimated table 2 could be reaches to other relations of E in refs. [80,81] for the selected values of G,η,α.

$R_{i}=R$ and $H={\sum_{J=2,}^{N}\left(\frac{1}{r_{1,j}}\right)}^{-1}$, for .i,j=1,2,⋯,N

and i≠j, moreover, due to the cavitation microbubbles have the same dynamics; H reveals to the distance between the centers of cavitation microbubble: N-cavitation microbubbles number. For that, we have

$$P_{proposed}\to \frac{\rho_{l}}{H}\left(N-1\right)\frac{d}{\mathrm{dt}}\left(R^{2}\frac{\mathrm{dR}}{\mathrm{dt}}\right)$$

The extended KME [14] of multi cavitation microbubbles dynamics during histotripsy under the effect of variable surface tension in the form

(11) $$\left(1-\frac{\dot{R}}{C}\right)R\ddot{R}+\frac{3}{2}\left(1-\frac{\dot{R}}{3C}\right){\dot{R}}^{2}=\frac{1}{\rho_{l}}\;\left(1+\frac{\dot{R}}{C}\right)\left\{P_{b}-P_{\infty }-2\frac{\sigma (t)}{R}+\frac{1}{2}G\left(3\alpha -1\right)\left(5-4\left(\frac{R_{0}}{R}\right)-4{\left(\frac{R_{0}}{R}\right)}^{4}\right)+2G\alpha \left(\frac{27}{40}+\frac{1}{8}{\left(\frac{R_{0}}{R}\right)}^{8}+\frac{1}{5}{\left(\frac{R_{0}}{R}\right)}^{5}+{\left(\frac{R_{0}}{R}\right)}^{2}-2\frac{R_{0}}{R}\right)-4\eta \frac{\dot{R}}{R}\right\}+\frac{1}{\rho_{l}}\frac{R}{c}\left\{-{3\kappa P_{g0}}_{\infty }{\left(\frac{R_{0}}{R}\right)}^{3\kappa }\frac{\dot{R}}{R}-\frac{d\sigma \left(t\right)}{\mathrm{dt}}-4\eta \left(\frac{R\ddot{R}-{\dot{R}}^{2}}{R^{2}}\right)+\frac{1}{2}G\left(3\alpha -1\right)\left(4\frac{R_{0}\dot{R}}{R^{2}}+4{\left(\frac{R_{0}}{R}\right)}^{4}\frac{\dot{R}}{R}\right)-2G\alpha \left({\left(\frac{R_{0}}{R}\right)}^{8}\frac{\dot{R}}{R}+{\left(\frac{R_{0}}{R}\right)}^{5}\frac{\dot{R}}{R}+2{\left(\frac{R_{0}}{R}\right)}^{2}\frac{\dot{R}}{R}+2\frac{\dot{R}}{R}\right)\right\}-\frac{1}{H}\left(N-1\right)\frac{d}{\mathrm{dt}}\left(R^{2}\frac{\mathrm{dR}}{\mathrm{dt}}\right)$$

here, it is remarkable that the surface tension σ(t) is the function of time. In the problem of fluid dynamics, we cannot neglect the study of variations effect of surface tension especially, on the cases of higher initial superheating or saturation, Authors [14] assumed that the definition of variable of the surface tension give us the better agreements with experimental data [84] as

(12) $$\sigma \left(t\right)=\rho_{l}gB_{2}R^{2}$$

where g is gravity. $B_{2}$ is the constant, can be estimated based on initial conditions. Authors [14,85] supposed the general formulae of variable surface tension as

(13) $$\sigma \left(t\right)=\rho_{l}gB_{m_{1}}R^{m_{1}}$$

where $m_{1}$ is the index; defines the generalized surface tension. Based on the Eqs. (13), (11), the modified of cavitation multi-bubbles model respect to the generalized surface tension becomes

(14) $$\left(1-\frac{\dot{R}}{C}\right)R\ddot{R}+\frac{3}{2}\left(1-\frac{\dot{R}}{3C}\right){\dot{R}}^{2}=\frac{1}{\rho_{l}}\;\left(1+\frac{\dot{R}}{C}\right)\left\{\left(bc^{n}+℧\left(C_{B}-C_{\infty }\right)\right)-2\rho_{l}gB_{m_{1}}R^{m_{1}-1}+\frac{1}{2}G\left(3\alpha -1\right)\left(5-4\left(\frac{R_{0}}{R}\right)-4{\left(\frac{R_{0}}{R}\right)}^{4}\right)+2G\alpha \left(\frac{27}{40}+\frac{1}{8}{\left(\frac{R_{0}}{R}\right)}^{8}+\frac{1}{5}{\left(\frac{R_{0}}{R}\right)}^{5}+{\left(\frac{R_{0}}{R}\right)}^{2}-2\frac{R_{0}}{R}\right)-4\eta \frac{\dot{R}}{R}\right\}+\frac{1}{\rho_{l}}\frac{R}{c}\left\{-{3\kappa P_{g0}}_{\infty }{\left(\frac{R_{0}}{R}\right)}^{3\kappa }\frac{\dot{R}}{R}-\frac{d\sigma \left(t\right)}{\mathrm{dt}}-4\eta \left(\frac{R\ddot{R}-{\dot{R}}^{2}}{R^{2}}\right)+\frac{1}{2}G\left(3\alpha -1\right)\left(4\frac{R_{0}\dot{R}}{R^{2}}+4{\left(\frac{R_{0}}{R}\right)}^{4}\frac{\dot{R}}{R}\right)-2G\alpha \left({\left(\frac{R_{0}}{R}\right)}^{8}\frac{\dot{R}}{R}+{\left(\frac{R_{0}}{R}\right)}^{5}\frac{\dot{S}}{S}+2{\left(\frac{R_{0}}{R}\right)}^{2}\frac{\dot{R}}{R}+2\frac{\dot{R}}{R}\right)\right\}-\frac{1}{H}\left(N-1\right)\frac{d}{\mathrm{dt}}\left(R^{2}\frac{d^{2}R}{dt^{2}}+2R{\left(\frac{\mathrm{dR}}{\mathrm{dt}}\right)}^{2}\right)$$

The details of the solution of modified of cavitation multi-bubbles model respect to the generalized surface tension was introduced in ref. [14], the solution of the behaviour of cavitation multibubble and the generalized solution of surface tension in multi-cavitation microbubbles in histotripsy becomes

(15) $$R\left(t\right)=\frac{Z_{1}}{{℧}_{4}}\left[Z_{2}+{℧}_{3}-2\rho_{l}gB_{m_{1}}R_{0}^{m_{1}-1}\Psi_{m}^{\frac{1}{3}\left(m_{1}-1\right)}+Z_{3}\left\{Z_{4}-\frac{2}{3\Phi_{0}}\rho_{l}gB_{2}R_{0}^{2}\Psi_{m}^{'}\right\}+Z_{6}-\frac{\left(N-1\right)}{H}\left(\frac{1}{3}\left(\Psi_{m}^{\frac{8}{3}}\Psi_{m}^{"}+\frac{2}{3}\Psi_{m}^{\frac{5}{3}}{\overset{´}{\Psi }}_{m}^{2}\right)+\frac{2}{9}S_{0}\Psi_{m}^{\frac{5}{3}}{\overset{´}{\Psi }}_{m}^{2}\right)\right]t^{\frac{1}{2}},$$

and

(16) $$\sigma \left(t\right)=\rho_{l}gB_{2}{{\left(\frac{Z_{1}}{{℧}_{4}}\left[\left\{Z_{2}+{℧}_{3}-2\rho_{l}gB_{m_{1}}R_{0}^{m_{1}-1}\Psi_{m}^{\frac{1}{3}\left(m_{1}-1\right)}\right\}+Z_{3}\left\{Z_{4}-\frac{2}{3\Phi_{0}}\rho_{l}gB_{2}{\mathrm{SR}}_{0}^{2}\Psi_{m}^{\prime }\right\}+Z_{6}-\frac{\left(N-1\right)}{H}\left(\frac{1}{3}\left(\Psi_{m}^{\frac{8}{3}}\Psi_{m}^{"}+\frac{2}{3}\Psi_{m}^{\frac{5}{3}}{\overset{´}{\Psi }}_{m}^{2}\right)+\frac{2}{9}R_{0}\Psi_{m}^{\frac{5}{3}}{\overset{´}{\Psi }}_{m}^{2}\right)\right]\right)}^{m_{1}}t}^{\frac{m_{1}}{2}}$$

All constants in Eqs. (15,16) are stated in ref. [14,38,72,84].

**Fig. 4:**
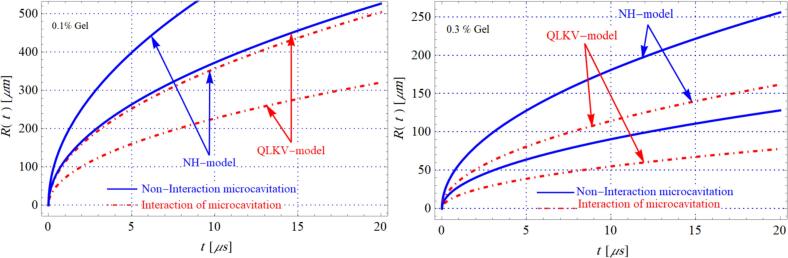
The dynamics of microbubble R(t) vs time t for 1%and2% of gel concentration N=3,H=5μm, G=200kPa, η=0.077Pas, $m_{1}=2$. Results published in [47].

The main results of significant role variable surface tension in single and multi-cavity bubbles are shown in Fig. 4. We found that variable surface tension helps suppression the growth processes of microcavitation bubbles rather than in the case of a single microbubbles, non-interaction microbubbles (N=1) and multi cavitation microbubbles (N>1) during histotripsy undertaking the impact of viscoelastic tissue. The impact of wide variety of cavitation microbubbles N at the cavitation dynamics for the duration of histotripsy has been proven in Fig. 4 wherein it's determined that the behaviour of cavitation microbubbles increase the wide variety of cavitation microbubbles decreases. Authors [14] determined that the changes in microbubbles are growing while the space among the microbubbles rates for the duration of the cavitation at the system of histotripsy are increasing. From those obtained results, the authors get that the space among the microbubble facilities complements the rates of microbubble dynamics. Fig. 4 indicates the position of gel concentration on cavitation non-interplay and interplay microbubble, wherein we get first that the conduct of microbubbles in gel concentration 0.1 % is bigger than in 0.3 %. For those results, the lower gel concentration considerably complements the cavitation microbubbles for the duration of the histotripsy. Moreover, we expect that gel concentration has an impact on the development of cavitation bubbles. The growth process at 0.1 % gel concentration is faster than the corresponding rates in the case at 0.3 % gel concentration. For this result, the impact of increasing gel concentration in viscoelastic biotissue reduces cavitation growth rates during the histotripsy [47].

The theoretical modeling of microcavitation dynamics presented in this section offers significant implications for the development and optimization of histotripsy-based therapeutic modalities. In histotripsy, high-intensity focused ultrasound (HIFU) initiates the rapid nucleation and violent collapse of cavitation bubbles, producing highly localized mechanical stresses that result in precise, non-thermal tissue fractionation. The analytical results derived in this study quantifying key parameters such as oscillation amplitude, stress wave propagation, and collapse timing across varying tissue stiffness and acoustic field conditions enable accurate estimation of the mechanical dose necessary for effective disruption of pathological tissue, while minimizing collateral damage to adjacent healthy structures. These findings are particularly relevant to clinical interventions involving non-invasive tumor ablation, targeted thrombolysis, and the removal of fibrotic or necrotic tissue. Moreover, the insights provided by the model contribute to the refinement of image-guided histotripsy protocols, by establishing quantitative relationships between acoustic input parameters and bubble behavior observable via ultrasound imaging. Such integration enhances real-time feedback control during treatment, ultimately improving procedural precision, safety, and therapeutic efficacy.

## 5 Lipid-encapsulated microbubbles in cells membranes

The cells membranes of lipid-coated symmetrical, single, and multi-bubbles are interesting in the field of viscoelastic and soft tissue. In this part, we aim to study the mechanism of membranes of lipid-coated microscopic cavitation with different approaches of bubbles behaviour in viscoelastic tissue, with considering the influences of lipid-shells and viscoelastic systems. A lipid-coated microbubble has been substantially utilized in numerous therapeutic, biological and biomedical applications [[86], [87], [88], [89]], that's one the class of agents in keeping with therapeutic, curing and diagnostic applications.

A lipid-coated microbubble in cells membranes may be filled with gas and created due to high-depth waves propagating throughout the medium and acoustic microcavitation. The scale of microbubbles is the size of a nano or micron that the variety of bubbles among 1:10 µm is appropriate for intravenous injection [90]. These microbubbles are applied as assessment agents withinside the scientific medication for growing or collapsing diagnostic imaging because there is an effect on larger through the tissues [87].

### 5.1 Mechanism of lipid-coated interaction microcavitations in viscoelastic tissues

**Fig. 5:**
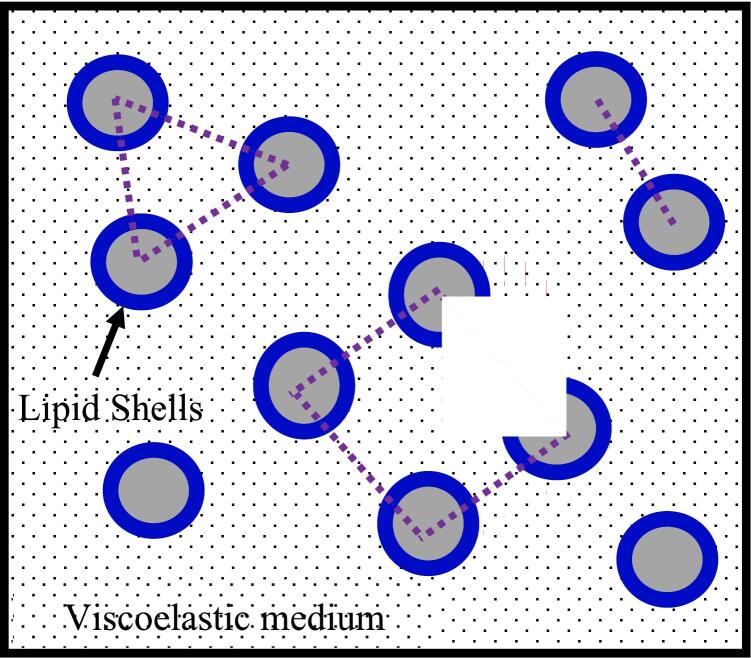
The system of lipid-coated non-interacting and interacting microbubbles under effect of viscoelastic medium, dashed-line represents $r_{\mathrm{ij}}$ which is the distance between two i-microbubble and j-bubble.

In this section, the study of lipid-coated membrane of multi microbubbles is important in the field of biological and medical problems as shown in Fig. 5. Many researchers are concerned about studying the membranes of lipid-coated microbubbles experimentally and theoretically. The theoretical and mathematical procedures of lipid-coated multi- cavitation microbubbles [21,91] were proposed in the different types of viscoelastic tissues for the duration of microscopic bubbles process. In this part, we introduce the investigation of two systems of lipid-coated bubbles as shown in a system of a lipid-coated single cavitation microbubble, and a system of multi-cavitation microbubbles. The two systems of the lipid-coated single and multi- cavitation microbubbles happen with considering the impact of constant and variable surface tension. It is considered that the membranes of lipid shells and viscoelastic tissues are considered in establishing and investigation the growth or collapse of lipid-coated multi microbubbles. The proposed models of lipid-coated cavitations reply to some biological and physical configurations such as number of microbubbles, bubble–bubble interactions, elasticity modulus, Van der Waals hard-core radius, polytropic exponent "κ" and others for single and multiple microbubbles.

The distance between microbubbles is $r_{\mathrm{ij}}$ between i- microbubble and j- microbubble. It was assumed the impact of surface tension at microbubble radius [19,21,22,92] as:

(17) $$\sigma \left(R\right)=\left\{\begin{array}{c} 0ifR\leq R_{\mathrm{bu}},\\ \chi \left(\frac{R^{2}}{R_{\mathrm{bu}}^{2}}-1\right)ifR_{\mathrm{bu}}<R<R_{\mathrm{break}-up},\\ \sigma_{\mathrm{tissue}}ifrupturedandR\geq R_{\mathrm{ru}}. \end{array}\right)$$

here χ is the of the lipid monolayer elasticity. $\sigma_{\mathrm{tissue}}$ refers to the surface tension in the biotissue (viscoelastic medium). $R_{\mathrm{bu}}$ refers to the buckling radius at the lower limit of the radius, which the lipid-coated shell is related to buckling. $R_{\mathrm{break}-up}$ refers to the upper limit of the microbubble’s radius. It is noted that among the buckling microbubble radius and break-up microbubble radius in the processes of lipid shell, there exists the shell surface elasticity. The values of χ, $R_{\mathrm{bu}}$ and $R_{\mathrm{break}-up}$ were presented in refs. [19,23].

The mathematical model of lipid-coated microbubble was described by continuity equation incompressible fluid, KME of microbubbles, and concentration equation [[93], [94], [95], [96]]. The model can put in spherical coordinates as:

(18) $$\frac{1}{\rho_{l}}\frac{\partial \rho }{\partial \;t}=-div\;\left(u\left(r,t\right)\right)$$

(19) $$\rho_{l}\left({\left(1-\frac{{\dot{R}}_{i}}{c}\right)R}_{i}{\ddot{R}}_{i}+{\left(\frac{3}{2}-\frac{\dot{R_{i}}}{2c}\right)\dot{R}}_{i}^{2}\right)=\left(1+\frac{{\dot{R}}_{i}}{c}\right)P_{\mathrm{tot},i}\left(t\right)+\frac{R_{i}}{c}\frac{d}{\mathrm{dt}}\left(P_{\mathrm{tot},\;i}\left(t\right)\right)$$

(20) $$\frac{1}{r^{2}}\;\frac{\partial }{\partial r}\left(r^{2}\frac{\partial C(r,t)}{\partial r}\right)=\frac{1}{a_{l}}\left(\frac{\partial C(r,t)}{\partial t}+u_{r}\frac{\partial C(r,t)}{\partial r}\right)$$

Based on the ref. [93,97], the pressure due to the interparticle interaction effect of microbubbles can be written as

(21) $${\mathrm{grad}\;P}_{j}=\frac{\;\rho_{l}}{r^{2}}\left(R_{j}^{2}\frac{d^{2}R_{j}}{dt^{2}}+2R_{j}\;{\left(\frac{dR_{j}}{\mathrm{dt}}\right)}^{2}\right)-\frac{2}{r^{5}}R_{j}^{4}{\left(\frac{dR_{j}}{\mathrm{dt}}\right)}^{2}=\frac{\rho_{l}}{r_{j}}\frac{d}{\mathrm{dt}}\left(R_{j}^{2}{\left(\frac{dR_{j}}{\mathrm{dt}}\right)}^{2}\right)+O\left(\frac{1}{r_{j}^{4}}\right)\;$$

The extended KME for lipid-coated multi-cavitation microbubbles for i- bubble and j- bubble as

(22) $$\rho_{l}\;\left({\left(1-\frac{{\dot{R}}_{i}}{c}\right)R}_{i}{\ddot{R}}_{i}+{\left(\frac{3}{2}-\frac{\dot{R_{i}}}{2c}\right)\dot{R}}_{i}^{2}\right)=\left(1+\frac{{\dot{R}}_{i}}{c}\right)P_{\mathrm{tot},i}\left(t\right)+\frac{R_{i}}{c}\frac{d}{\mathrm{dt}}\left(P_{\mathrm{tot},\;i}\left(t\right)\right)-\frac{\rho_{l}}{r_{\mathrm{ij}}}\sum_{J=1,i\neq j}^{n}\frac{d}{\mathrm{dt}}\left(R_{j}^{2}\frac{dR_{j}}{\mathrm{dt}}\right)$$

In the case of balance of typical stress at the microbubble interface, the total pressure and gas pressure inside microbubbles [[93], [94], [95], [96],^98^] can be stated as

(23) $${P_{\mathrm{tot},\;i}\left(t\right)=P}_{g}\left(t\right)-P_{0}-\frac{2\sigma \left(R\right)}{R_{i}}+{\left(\tau_{\mathrm{rr}}\right|}_{r=R}-\frac{4k_{s}\dot{R}}{R^{2}}$$

(24) $$P_{g}\left(t\right)=\left(P_{0}+\frac{2\sigma_{0}\left(R_{i0}\right)}{R_{i0}}\right){\left(\frac{R_{i0}^{3}-h_{i}^{3}}{R_{i}^{3}-h_{i}^{3}}\right)}^{\kappa },\;k_{s}=\frac{k_{0}R}{R+\alpha \left|\dot{R}\right|}$$

here $k_{s}$ refers to a shell viscosity in the study of liposome [67]. And the shear stress at the cavitation bubble interfaces [65,66] can be put as

(25) $${\left(\tau_{\mathrm{rr}}\right|}_{r=R}=-\left(\frac{4G\left({R^{3}-R}_{0}^{3}\right)}{3R^{3}}+4\eta \frac{\dot{R}}{R}\right)$$

Combining Eqs. (18–25), the extended KME of cavitation microbubble behaviors in a membrane of a lipid-coated system reduces to

(26) $$\rho_{l}\;\left({\left(1-\frac{{\dot{R}}_{i}}{c}\right)R}_{i}{\ddot{R}}_{i}+{\left(\frac{3}{2}-\frac{{\dot{R}}_{i}}{2c}\right)\dot{R}}_{i}^{2}\right)=\left(1+\frac{{\dot{R}}_{i}}{c}\right)\left[P_{g,i}\left(t\right)-P_{0}\right]-\left(\left(1+\frac{{\dot{R}}_{i}}{c}\right)\frac{2\sigma \left(R\right)}{R_{I}}+\frac{R_{i}}{c}\frac{d}{\mathrm{dt}}\left(\frac{2\sigma \left(R\right)}{R_{I}}\right)\right)-3\frac{\kappa }{c}\left(P_{0}+\frac{2\sigma_{0}\left(R_{i0}\right)}{R_{i0}}\right)R_{i}^{3}\dot{R}\frac{{\left(R_{i0}^{3}-h_{i}^{3}\right)}^{\kappa }}{{\left(R_{i}^{3}-h_{i}^{3}\right)}^{\kappa +1}}-4G\frac{R_{0}^{3}\dot{R}}{3R^{3}}\left(\left(R^{2}-R_{0}^{2}\right)+\frac{\dot{R_{i}}}{c}\left(R^{2}-2R_{0}^{2}\right)\right)-4\mu \frac{{\dot{R}}_{i}}{R_{i}}\left(1+\frac{\ddot{R}R}{c{\dot{R}}_{i}}\right)-4k_{0}\left[\left(1+\frac{{\dot{R}}_{i}}{c}\right)\frac{R_{i}{\dot{R}}_{i}}{R_{i}+\alpha {\dot{R}}_{i}}+\frac{\ddot{R}R^{2}-{\dot{R}}_{i}^{2}\left(2R_{i}+\alpha {\dot{R}}_{i}\right)}{{\mathrm{cR}}_{i}{\left({\;R}_{i}+\alpha {\dot{R}}_{i}\right)}^{2}}\right]-\frac{\rho_{l}}{r_{\mathrm{ij}}}\sum_{J=1,i\neq j}^{n}\frac{d}{\mathrm{dt}}\left(R_{j}^{2}\frac{dR_{j}}{\mathrm{dt}}\right)$$

The initial and boundary conditions for a microbubble are defined as:

(27) $$R\left(t_{0i}\right)=R_{0i},\;\frac{dR(t_{0i})}{dt}={\dot{R}}_{0i},\;and\;\frac{d^{2}R\left(t_{0i}\right)}{dt^{2}}=\;0,\;C_{R_{i}}\left(t_{0i}\right)=C_{0i}$$

(28) $$R\left(t_{m_{i}}\right)=R_{\mathrm{fi}},\;\frac{dR(t_{f_{i}})}{dt}={\dot{R}}_{m_{i}},\;and\;\frac{d^{2}R\left(t_{f_{i}}\right)}{dt^{2}}=\;0$$

It is observed that the Eq. (26) can be solved analytically using the initial and boundary conditions of Eqs. (27–28) and applying the PZA [43] for single and multiple lipid-coated cavitation bubbles, as summarized in Table 3, where it is obtained that the radius lipid-coated cavitation microbubbles for different values of viscoelastic medium is stated for non-interaction microbubbles as single microbubble and interparticle interaction of microbubbles.

**Table 3:** Results of lipid-coated cavitation microbubbles for different values of viscoelastic medium.

Model of lipid coated cavitation	Type of surface tension	The radius of lipid-coated cavitation bubble	Type of solution method	Formulae of lipid shells
Single microbubbles	$\sigma_{\mathrm{tissue}}$ is a const**.**	$R\left(t\right)=\frac{6\left(c+{\dot{R}}_{0}\right)\sigma }{R_{0}\;\beta_{1}\left(3C+\gamma R_{0}\Phi_{0}^{-\frac{2}{3}}\;\overset{´}{S_{f}}\right)}{\left({\frac{12}{\pi }a}_{l}\;\right)}^{\frac{1}{2}}\left\{\frac{\left(C+\frac{1}{3}\gamma R_{0}\;\Phi_{0}^{-\frac{2}{3}}\;{\overset{´}{S}}_{f}\right)}{\rho_{l}\left(c+{\dot{R}}_{0}\right)\gamma^{2}R_{0}^{2}}\beta_{1}-\frac{2\sigma_{\mathrm{tiss}}\Phi_{0}^{\frac{1}{3}}}{\rho_{l}\gamma^{2}R_{0}^{3}}-\left({\frac{\kappa }{\rho c}\Gamma }_{1}+{\frac{4}{3}G\Gamma }_{2}+{\frac{4}{3}\mu \phi_{0}^{-\frac{1}{3}}\Gamma }_{3}+{4k_{0}\Gamma }_{4}\right)\right\}t^{\frac{1}{2}}$	Plesset-Zwick Method [10]	$\mathrm{LCS}=\frac{2\;\sigma_{\mathrm{tiss}}}{Z_{2}\sqrt{t}}+\frac{4\;k_{0}}{Z_{2}^{2}\left(t+\frac{1}{2}\alpha \right)}$.
Single microbubbles	$\sigma \left(R\right)=\chi \left(\frac{R^{2}}{R_{\mathrm{bu}}^{2}}-1\right)$	$R\left(t\right)=\frac{6\left(c+{\dot{R}}_{0}\right)\sigma 1}{R_{0}\;\beta_{2}\left(3C+\gamma_{1}R_{0}\;\Phi_{0}^{-\frac{2}{3}}\;\overset{´}{S_{f}}\right)}{\left({\frac{12}{\pi }a}_{l}\;\right)}^{\frac{1}{2}}\left\{\frac{\left(C+\frac{1}{3}\gamma_{1}R_{0}\;\Phi_{0}^{-\frac{2}{3}}\;{\overset{´}{S}}_{f}\right)}{\rho_{l}\left(c+{\dot{R}}_{0}\right)\gamma_{1}^{2}R_{0}^{2}}\beta_{2}-\frac{2\chi }{\gamma_{1}^{2}R_{0}^{2}}\left(\frac{R_{0}\Phi_{0}^{-\frac{1}{3}}}{R_{\mathrm{bu}}^{2}}\left(1+\frac{2}{3}\frac{{\gamma_{1}R}_{0}\;\Phi_{0}^{-\frac{2}{3}}\;\overset{´}{S_{f}}}{c}\right)-\frac{\Phi_{0}^{\frac{1}{3}}}{R_{0}}\right)-\left(\frac{\kappa }{\rho c}\Gamma_{5}+\frac{4}{3}\left({G\Gamma }_{6}+{\Phi_{0}^{-\frac{1}{3}}\mu \Gamma }_{7}\right)+4k_{0}\Gamma_{8}\right)\right\}t^{\frac{1}{2}}$.	Plesset-Zwick Method [10]	$\mathrm{LCS}=\frac{2\chi \;\left(Z_{1}^{2}t-R_{\mathrm{bu}}^{2}\right)}{Z_{1}\;R_{\mathrm{bu}}^{2}\sqrt{t}}+\frac{4\;k_{0}}{Z_{2}^{2}\left(t+\frac{1}{2}\alpha \right)}$.
Multiple microbubbles	$\sigma_{\mathrm{tissue}}$ is a const.	$R\left(t\right)=\frac{6\left(c+{\dot{R}}_{0}\right)\sigma 1}{R_{0}\;\beta_{4}\left(3C+\gamma R_{0}\;\Phi_{0}^{-\frac{2}{3}}\;\overset{´}{S_{f}}\right)}{\left({\frac{12}{\pi }a}_{l}\;\right)}^{\frac{1}{2}}\left\{\frac{\left(C+\frac{1}{3}\gamma_{1}R_{0}\;\Phi_{0}^{-\frac{2}{3}}\;{\overset{´}{S}}_{f}\right)}{\rho_{l}\left(c+{\dot{R}}_{0}\right)\gamma_{1}^{2}R_{0}^{2}}\beta_{4}-\frac{2\chi }{\gamma_{1}^{2}R_{0}^{2}}\left(\frac{R_{0}\Phi_{0}^{-\frac{1}{3}}}{R_{\mathrm{bu}}^{2}}\left(1+\frac{2{\gamma_{1}R}_{0}\;\Phi_{0}^{-\frac{2}{3}}\;\overset{´}{S_{f}}}{3c}\right)-\frac{\Phi_{0}^{\frac{1}{3}}}{R_{0}}\right)-\left(\frac{\kappa }{\rho c}\Gamma_{5}+\frac{4}{3}\left({G\Gamma }_{6}+{\Phi_{0}^{-\frac{1}{3}}\mu \Gamma }_{7}\right)+4k_{0}\Gamma_{8}\right)-\;\frac{\left(N-1\right)}{H}\Phi_{0}^{-\frac{5}{3}}\left(\frac{1}{3}\left(\frac{S_{f}^{"}}{\Phi_{0}}+\frac{2}{3}{\overset{´}{S}}_{f}^{2}\right)+\frac{2}{9}R_{0}^{3}{\overset{´}{\;S}}_{f}^{2}\right)\right\}t^{\frac{1}{2}}$.	Plesset-Zwick Method [43]	$\mathrm{LCS}=\frac{2\;\sigma_{\mathrm{tiss}}}{Z_{2}\sqrt{t}}+\frac{4\;k_{0}}{Z_{2}^{2}\left(t+\frac{1}{2}\alpha \right)}$
Multiple microbubbles	$\sigma \left(R\right)=\chi \left(\frac{R^{2}}{R_{\mathrm{bu}}^{2}}-1\right)$	$R\left(t\right)=\frac{6\left(c+{\dot{R}}_{0}\right)\sigma 1}{R_{0}\;\beta_{4}\left(3C+\gamma R_{0}\;\Phi_{0}^{-\frac{2}{3}}\;\overset{´}{S_{f}}\right)}{\left({\frac{12}{\pi }a}_{l}\;\right)}^{\frac{1}{2}}\left\{\frac{\left(C+\frac{1}{3}\gamma_{1}R_{0}\;\Phi_{0}^{-\frac{2}{3}}\;{\overset{´}{S}}_{f}\right)}{\rho_{l}\left(c+{\dot{R}}_{0}\right)\gamma_{1}^{2}R_{0}^{2}}\beta_{4}-\frac{2\chi }{\gamma_{1}^{2}R_{0}^{2}}\left(\frac{R_{0}\Phi_{0}^{-\frac{1}{3}}}{R_{\mathrm{bu}}^{2}}\left(1+\frac{2}{3}\frac{{\gamma_{1}R}_{0}\;\Phi_{0}^{-\frac{2}{3}}\;\overset{´}{S_{f}}}{c}\right)-\frac{\Phi_{0}^{\frac{1}{3}}}{R_{0}}\right)-\left(\frac{\kappa }{\rho c}\Gamma_{5}+\frac{4}{3}\left({G\Gamma }_{6}+{\Phi_{0}^{-\frac{1}{3}}\mu \Gamma }_{7}\right)+4k_{0}\Gamma_{8}\right)-\;\frac{\left(N-1\right)}{H}\Phi_{0}^{-\frac{5}{3}}\left(\frac{1}{3}\left(\frac{S_{f}^{"}}{\Phi_{0}}+\frac{2}{3}{\overset{´}{S}}_{f}^{2}\right)+\frac{2}{9}R_{0}^{3}{\overset{´}{\;S}}_{f}^{2}\right)\right\}t^{\frac{1}{2}}$	Plesset-Zwick Method [43]	$\mathrm{LCS}=\frac{2\chi \;\left(Z_{1}^{2}t-R_{\mathrm{bu}}^{2}\right)}{Z_{1}\;R_{\mathrm{bu}}^{2}\sqrt{t}}+\frac{4\;k_{0}}{Z_{2}^{2}\left(t+\frac{1}{2}\alpha \right)}$.

Additionally, we can estimate lipid-coated shells based on the mechanism of cavitation microbubble, where the lipid-coated shells (LCS) refer to the summation of shell elasticity and shell viscosity in mono or multi-layer, where was taken this relation below and Table 3 as

(29) $$\mathrm{LCS}=\frac{2\sigma \left(R\right)}{R}+\frac{4k_{s}\dot{R}}{R^{2}}.$$

It is found that the numerical results of lipid shells in Eq. (29) are revealed in Table 3 (these results are in ref. [93]) where, authors introduced the comparison results of LCS between the non-interaction microbubbles at (N=1) and multiple microbubbles at (n≥2) for constant and variable surface tension. We conclude that the LCS it is inversely proportional to the increase in the number of microbubbles. Moreover, the LCS it is inversely related to the time during the lipid-coated cavitation growth process. The impact of variable surface tension will be taken into account during the dynamics of lipid-shell single and multiple bubbles. Also, the translational motions of multiple microbubbles and inertia forces are ignored. The splitting of microscopic cavitation bubbles is not considered. The process of lipid-coated bubbles happens in viscoelastic tissue via considering the initial of microbubbles at $t_{0i}>0,$ $R\left(t_{0i}\right)=R_{0i}>0$ and $\frac{dR(t_{0i})}{dt}={\dot{R}}_{0i}$; i=1,2,3,⋯,N where parameter "N" is the bubbles number of microbubbles; N≥1.

All constants in the above table are defined and mentioned in ref. [21].

The number of bubbles "N" and other parameters like impacts of bubble–bubble interparticle interactions, elasticity modulus, Van der Waals hard-core radius, polytropic exponent "κ" are studied on growing and collapsing of dynamics of lipid-shell bubbles in viscoelastic tissue. It is expected that results relate to be the lipid-coated microbubbles via considering the impacts of variation of surface tension σ(R) of biotissue. Additionally, the results are interesting in the clarification and appearance of the problem of lipid-coated bubbles in different types of viscoelastic tissue.

It is clear that the growth or collapse of multiple cavitation microbubbles in the case of constant surface tension is higher than in the case of variable one. The analytical results of lipid-coated multiple microbubbles agree with the results of the physical approaches in ref. [99,100] in the stage of collapse in a Newtonian fluid.

### 5.2 Impact of lipid shell thickness on multiple encapsulated cavitation microbubbles

Encapsulated cavitation microbubbles are empty, nano- or micro-meter sized, symmetric spherical bubbles [101,102] which are utilized in several therapeutic, biological and biomedical applications with effect of ultrasound such as cell membranes of lipid-shells, lithotripsy, histotripsy, and diver’s tissue [[101], [102], [103], [104], [105], [106], [107], [108], [109], [110]]. In order to increase and enhance the difference rates between the blood pool and biotissue, encapsulated cavitation microbubbles had been employed as ultrasound or ultrasonic contrast medium in experimental works and clinical diagnosis.

The objective of this section is to study the behavior of lipid-encapsulated single cavitation microbubble and multiple cavitation microbubbles in soft tissues, like an examination of the impact of the thickness of the lipid-encapsulated microbubbles envelope located between the inner and outer radius of the microbubble theoretically and analytically based on mathematical and physical concepts. Besides, the proposed models of modified Church equations were solved analytically by using the modified PZA taking into account the initial time of bubbles growth. Although ultrasound and encapsulated microbubbles have been extensively used in a variety of medical fields, however little is known about the dynamics of the bubbles in the viscoelastic medium. Whereas the coated microbubbles and the nature of the medium surrounding them all play intricate roles in many different industrial and medical applications. Additionally, authors [101,[111], [112], [113]] investigated the factors affecting the growth behaviour of microbubble dynamics of different materials such as polymers, albumin, blood, lipids, and liquids (as shown in figures in ref. [101]). Besides that, our obtained results for single microbubbles (N=1) and multiple encapsulated microbubbles (N≥2) were numerically compared and discussed with results in previous work [21]. The effect of strong and weak of thickness layers on encapsulated cavitation microbubbles in cell membranes are introduced on Table 4.

In the case of strongly shell thickness, the a viscoelastic medium under impact of shell thickness layer δ on single microbubble with initial inner and outer radii ($R_{a0}\;and\;R_{b0}$) could occur radial inner and outer over time t, i.e.,$\;R_{a0}$ and $R_{b0}$ respectivetly; $t>0,\;R_{a0}\geq 0$, $R_{b0}\geq 0$, the relation between inner radius of microbubble $R_{a}$ and outer radius $R_{b}$ under effect of shell thickness can be put as

$$R_{b}=R_{a}+\delta$$

In the situation of weak shell thickness, the limit δ→0, for the infinitesimal shell thickness, then δ reduces to infinitesimal as well. Physically, the shell thickness is very small but finite, it cannot be omitted. Suppose $R_{bi0}=R_{ai0}+\delta_{0i}$; i=1,2,3,⋯,N; $\delta_{0}$ is the shell thickness at rest. Assuming that $\delta_{0i}\ll R_{bi0}$, one has $R_{bi0}^{3}-R_{ai0}^{3}\approx 3\delta_{0}R_{0}^{2}$. The description of interaction microbubbles with influence of zero shell thickness in Table 4 which summarizes the mathematical models of encapsulated cavitation microbubbles in cell membranes under effect of thickness layers. Additionally, Table 4 gives us the solution of encapsulated cavitation models (i.e., radius of encapsulated cavitation microbubbles) under effect of variety of surface tension and other physical parameters such as Jacob number, viscosity, and so on.

**Table 4:** Summary of the given models of encapsulated cavitation microbubbles [100] in cell membranes under effect of thickness layers.

Type of theoretical model	Effect of thickness layers	Mathematical model	Results
Single microbubble	Strong	$R_{a}\frac{d^{2}R_{a}}{dt^{2}}\left\{1+\frac{1}{\rho_{s}}\left(\rho_{l}-\rho_{s}\right)\frac{R_{a}}{\left(R_{a}+\;\delta \right)}\right\}+{\left(\frac{dR_{a}}{\mathrm{dt}}\right)}^{2}\left\{\frac{3}{2}+\frac{1}{\rho_{s}}\left(\rho_{l}-\rho_{s}\right)\left(\frac{4{\left(R_{a}+\;\varepsilon \right)}^{2}-R_{a}^{3}}{2{\left(R_{a}+\;\delta \right)}^{3}}\right)\frac{R_{a}}{\left(R_{a}+\delta \right)}\right\}=\frac{1}{{\;\rho }_{s}}\left\{P_{G0}{\left(\frac{R_{a0}}{R_{a}}\right)}^{3\kappa }-\frac{2\sigma_{a}}{R_{a}}-\frac{2\sigma_{b}}{\left(R_{a}+\delta \right)}-4\eta_{l}\frac{R_{a}^{2}}{{\left(R_{a}+\delta \right)}^{3}}\frac{dR_{a}}{\mathrm{dt}}-P_{0}-P_{\mathrm{ac}}\left(t\right)-4\mu_{s}\left(\frac{R_{b0}^{3}-R_{a0}^{3}}{{\left(R_{a}+\;\delta \right)}^{3}}\right)\left(1-\frac{R_{1e}}{R_{a}}\right)-4\eta_{s}\left(\frac{R_{b0}^{3}-R_{a0}^{3}}{R_{a}{\left(R_{a}+\;\delta \right)}^{3}}\right)\frac{dR_{a}}{\mathrm{dt}}\right\}$	$R_{a}\left(t\right)=\frac{2R_{a0}^{2}\rho_{s}\Gamma^{2}}{{\pi E}_{2}}\left[\frac{R_{a0}\left(\rho_{s}-\rho_{l}\right)}{{3\rho }_{s}\left(R_{a}+\delta \right)}\Phi_{0}^{-\frac{2}{3}}\left(\Phi_{0}^{-2}X_{m}^{"}+\frac{2}{3}\Phi_{0}^{-1}\;{\overset{´}{X}}_{m}^{2}\right)+\frac{R_{a0}\left(\rho_{s}-\rho_{l}\right)}{18\rho_{s}}\frac{\left(4{\left(R_{a0}\Phi_{0}^{\frac{-1}{3}}+\;\delta \right)}^{2}-R_{a0}^{3}X\;\right)}{{\left(R_{a0}\Phi_{0}^{\frac{-1}{3}}+\delta \right)}^{4}}\Phi_{0}^{\frac{-5}{3}}\;{\overset{´}{X}}_{m}^{2}+\frac{1}{{\rho_{s}\Gamma }^{2}R_{a0}^{2}\rho_{s}}E_{2}-\frac{1}{{\;\Gamma^{2}R_{a0}^{2}\rho }_{s}}\left\{\frac{2\sigma_{a}}{R_{a0}}\Phi_{0}^{\frac{1}{3}}+\frac{2\sigma_{b}}{\left(R_{a0}\Phi_{0}^{\frac{-1}{3}}+\delta \right)}+\frac{4}{3}\eta_{l}\frac{1}{{\Gamma^{2}R_{a0}\left(R_{a0}\Phi_{0}^{\frac{-1}{3}}+\delta \right)}^{3}}\Phi_{0}^{\frac{-4}{3}}\;{\overset{´}{X}}_{m}+4\mu_{s}\left(\frac{R_{b0}^{3}-R_{a0}^{3}}{{\left(R_{a0}\Phi_{0}^{\frac{-1}{3}}+\;\delta \right)}^{3}}\right)\left(1-\frac{R_{1e}}{R_{a0}}\Phi_{0}^{\frac{1}{3}}\right)+\frac{4}{3}\eta_{s}\Gamma \left(\frac{R_{b0}^{3}-R_{a0}^{3}}{{\left(R_{a0}\Phi_{0}^{\frac{-1}{3}}+\;\delta \right)}^{3}}\right)\Phi_{0}^{\frac{-1}{3}}\;\overset{´}{X_{m}}\right\}\right]J_{a}{\left(\frac{12}{\pi }a_{l}t\right)}^{\frac{1}{2}}$
Multiple microbubbles	Strong	$R_{a}\frac{d^{2}R_{\mathrm{ai}}}{dt^{2}}\left\{1+\frac{1}{\rho_{s}}\left(\rho_{l}-\rho_{s}\right)\frac{R_{\mathrm{ai}}}{R_{\mathrm{bi}}}\right\}+{\left(\frac{dR_{\mathrm{ai}}}{\mathrm{dt}}\right)}^{2}\left\{\frac{3}{2}+\frac{1}{\rho_{s}}\left(\rho_{l}-\rho_{s}\right)\left(\frac{4R_{\mathrm{bi}}^{3}-R_{\mathrm{ai}}^{3}}{2R_{\mathrm{bi}}^{3}}\right)\frac{R_{\mathrm{ai}}}{R_{\mathrm{bi}}}\right\}=\frac{1}{\rho_{s}}\left\{P_{G0}{\left(\frac{R_{a0}}{R_{\mathrm{ai}}}\right)}^{3\kappa }-\frac{2\sigma_{a}}{R_{\mathrm{ai}}}-\frac{2\sigma_{\mathrm{bi}}}{R_{\mathrm{bi}}}-4\eta_{l}\frac{R_{\mathrm{ai}}^{2}}{R_{\mathrm{bi}}^{3}}\frac{dR_{\mathrm{ai}}}{\mathrm{dt}}-P_{0}-4\mu_{s}\left(\frac{R_{b0}^{3}-R_{a0}^{3}}{R_{\mathrm{bi}}^{3}}\right)\left(1-\frac{R_{1e}}{R_{a}}\right)-4\eta_{s}\left(\frac{R_{b0}^{3}-R_{a0}^{3}}{R_{\mathrm{ai}}R_{\mathrm{bi}}^{3}}\right)\frac{dR_{\mathrm{ai}}}{\mathrm{dt}}\right\}-\sum_{i\neq j,j=1}^{N}\frac{1}{s_{\mathrm{ij}}}\frac{d\left(R_{\mathrm{aj}}^{2}{\dot{R}}_{\mathrm{aj}}\right)}{\mathrm{dt}}$	$R_{a}\left(t\right)=\frac{2R_{a0}^{2}\rho_{s}\Gamma^{2}}{{\pi E}_{4}}\left[\frac{R_{a0}\left(\rho_{s}-\rho_{l}\right)}{{3\rho }_{s}\left(R_{a}+\delta \right)}\Phi_{0}^{-\frac{2}{3}}\left(\Phi_{0}^{-2}X_{m}^{"}+\frac{2}{3}\Phi_{0}^{-1}{\overset{´}{X}}_{m}^{2}\right)+\frac{R_{a0}\left(\rho_{s}-\rho_{l}\right)\left(4{\left(R_{a0}\Phi_{0}^{\frac{-1}{3}}+\delta \right)}^{2}-R_{a0}^{3}X\right)}{18\rho_{s}{\left(R_{a0}\Phi_{0}^{\frac{-1}{3}}+\delta \right)}^{4}}\Phi_{0}^{\frac{-5}{3}}{\overset{´}{X}}_{m}^{2}+\frac{1}{{\rho_{s}\Gamma }^{2}R_{a0}^{2}\rho_{s}}E_{2}-\frac{1}{{\Gamma^{2}R_{a0}^{2}\rho }_{s}}\left\{\frac{2\sigma_{a}}{R_{a0}}\Phi_{0}^{\frac{1}{3}}+\frac{2\sigma_{b}}{\left(R_{a0}\Phi_{0}^{\frac{-1}{3}}+\delta \right)}+\frac{4}{3}\eta_{l}\frac{1}{{\Gamma^{2}R_{a0}\left(R_{a0}\Phi_{0}^{\frac{-1}{3}}+\delta \right)}^{3}}\Phi_{0}^{\frac{-4}{3}}{\overset{´}{X}}_{m}+4\mu_{s}\left(\frac{R_{b0}^{3}-R_{a0}^{3}}{{\left(R_{a0}\Phi_{0}^{\frac{-1}{3}}+\delta \right)}^{3}}\right)\left(1-\frac{R_{1e}}{R_{a0}}\Phi_{0}^{\frac{1}{3}}\right)+\frac{4}{3}\eta_{s}\Gamma \left(\frac{R_{b0}^{3}-R_{a0}^{3}}{{\left(R_{a0}\Phi_{0}^{\frac{-1}{3}}+\delta \right)}^{3}}\right)\Phi_{0}^{\frac{-1}{3}}\overset{´}{X_{m}}\right\}-\frac{\left(N-1\right)}{S}\Phi_{0}^{\frac{-5}{3}}\left(\frac{1}{3}\Phi_{0}^{\frac{-1}{3}}X_{m}^{"}+\frac{2}{9}\left(R_{0}+2\right){\overset{´}{\;X}}_{m}^{2}\right)\right]J_{a}{\left(\frac{12}{\pi }a_{l}t\right)}^{\frac{1}{2}}$
Single microbubble	Weak	$R\frac{d^{2}R}{dt^{2}}+\frac{3}{2}{\left(\frac{\mathrm{dR}}{\mathrm{dt}}\right)}^{2}=\frac{1}{\rho_{l}}\left\{P_{g}-\frac{2\sigma }{R}-4\frac{\eta_{l}}{R}\frac{\mathrm{dR}}{\mathrm{dt}}-P_{0}-12\mu_{s}\left(\frac{\delta_{0}R_{0}^{2}}{R^{3}}\right)\left(1-\frac{R_{0}}{R}\right)-12\eta_{s}\left(\frac{\delta_{0}R_{0}^{2}}{R^{4}}\right)\frac{\mathrm{dR}}{\mathrm{dt}}\right\}$	$R\left(t\right)=\frac{2R^{2}\rho_{l}\Gamma^{2}}{{\pi E}_{9}}\left[\frac{1}{{R_{0}^{2}\Gamma^{2}\rho }_{l}}\left\{E_{8}-\frac{2\sigma }{R_{0}}\Phi_{0}^{\frac{1}{3}}-\frac{4}{3}\eta_{l}\Gamma \Phi_{0}^{\frac{-1}{3}}{\overset{´}{X}}_{m}-12\frac{\mu_{s}\delta_{0}}{R_{0}X}\left(1-\Phi_{0}^{\frac{1}{3}}\right)-4\frac{{\eta_{s}\delta }_{0}}{R_{0}}\Gamma \;\Phi_{0}^{\frac{2}{3}}{\overset{´}{X}}_{m}\right\}\right]J_{a}{\left(\frac{12}{\pi }a_{l}t\right)}^{\frac{1}{2}}$
Multiple microbubbles	Weak	$R\frac{d^{2}R}{dt^{2}}+\frac{3}{2}{\left(\frac{\mathrm{dR}}{\mathrm{dt}}\right)}^{2}=\frac{1}{\rho_{l}}\left\{P_{g}-\frac{2\sigma }{R}-4\frac{\eta_{l}}{R}\frac{\mathrm{dR}}{\mathrm{dt}}-P_{0}-12\mu_{s}\left(\frac{\delta_{0}R_{0}^{2}}{R^{3}}\right)\left(1-\frac{R_{0}}{R}\right)-12\mu_{s}\left(\frac{\delta_{0}R_{0}^{2}}{R^{4}}\right)\frac{\mathrm{dR}}{\mathrm{dt}}\right\}-\frac{\left(N-1\right)}{H}\frac{d\left(R^{2}\frac{\mathrm{dR}}{\mathrm{dt}}\right)}{\mathrm{dt}}$	$R\left(t\right)=\frac{2R^{2}\rho_{l}\Gamma^{2}}{{\pi E}_{9}}\left[\frac{1}{{R_{0}^{2}\Gamma^{2}\rho }_{l}}\left\{E_{8}-\frac{2\sigma }{R_{0}}\Phi_{0}^{\frac{1}{3}}-\frac{4}{3}\eta_{l}\Gamma \Phi_{0}^{\frac{-1}{3}}{\overset{´}{X}}_{m}-12\frac{\mu_{s}\delta_{0}}{R_{0}X}\left(1-\Phi_{0}^{\frac{1}{3}}\right)-4\frac{{\eta_{s}\delta }_{0}}{R_{0}}\Gamma \;\Phi_{0}^{\frac{2}{3}}{\overset{´}{X}}_{m}\right\}-\frac{\left(N-1\right)}{H}\Phi_{0}^{\frac{-5}{3}}\left[\frac{1}{3}\Phi_{0}^{-1}X_{m}^{"}+\frac{2}{9}\left(R_{0}+2\right){\overset{´}{\;X}}_{m}^{2}\right]\right]J_{a}{\left(\frac{12}{\pi }a_{l}t\right)}^{\frac{1}{2}}$

In conclusion, the obtained inner radius encapsulated symmetrical, single cavitation microbubble is more than the multiple of encapsulated cavitation microbubbles. The number of encapsulated N-bubbles reduces the behavior of inner and outer microbubbles in viscoelastic tissues. The obtained results agree significantly with the published results [38] in the case of collapse stage in a Newtonian fluid and theoretical approach [21] in viscoelastic medium in the stage of growth dynamics with weakly shell-thickness.

The theoretical insights into the dynamics of lipid-encapsulated microbubbles have direct relevance to a range of biomedical applications. In drug delivery, understanding the rupture and deformation thresholds of the lipid shell under varying acoustic pressures is essential for achieving controlled release at the cellular level. In particular, our modeling helps predict the critical pressure needed to induce permeabilization of the cell membrane without causing irreversible damage, which is vital in ultrasound-mediated drug and gene delivery strategies.

Additionally, the model provides a predictive framework for optimizing acoustic parameters in histotripsy, where controlled cavitation is used to fractionate tissue. In contrast-enhanced ultrasound imaging, the mechanical stability and oscillation behavior of lipid-shelled bubbles influence both signal enhancement and imaging resolution. Thus, the interplay between shell elasticity, viscosity, and surrounding tissue mechanics captured in our model offers valuable guidance for designing microbubbles with tailored properties for specific clinical needs.

## 6 Decompression illness based on the dynamics of gas bubbles in the biotissue

**Fig. 6:**
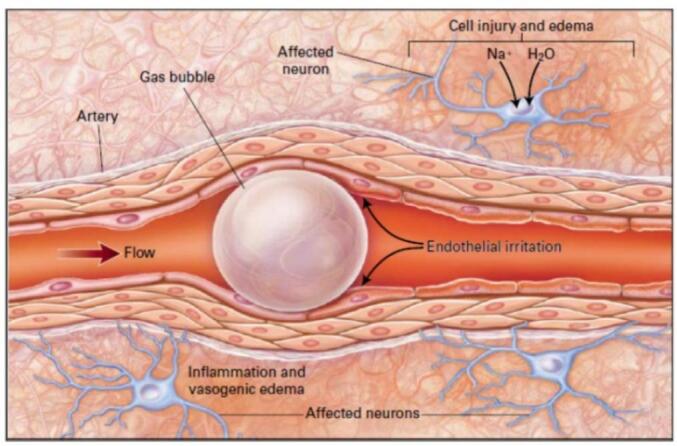
A bubble that blocks the flow of blood through a cerebral artery that ranges in diameter from 30 to 60 μm, leading to distal ischemia. Neuronal metabolic functions are disrupted by blockage [117].

A pathology known as decompression illness (DCI) affects compressed air workers, pilots, divers, and astronauts. It comes from a drop in ambient pressure brought on by bubbles that form in the body during or after decompression. Decompression sickness includes both arterial gas embolism and decompression sickness as illustrated in Fig. 6, it is found that it can be complicated to distinguish, know, and require the same treatment [[114], [115], [116]]. Arterial gas embolism is caused by gas emboli in the arterial circulation and may also have iatrogenic causes. Venous gas emboli that enter the arterial circulation or pulmonary overexpansion that tears alveolar capillaries can both produce these emboli. Decompression sickness, also colloquially referred to as “the bends,” is caused by the formation of bubbles from dissolved inert gas in the tissues during decompression.

Assume that a thin boundary layer, a well-stirred finite tissue, and the gas bubble can all be used to model the formation of a gas bubble within some tissue. The solvent gas pressure is ignored and only considers the diffusible gas in the expanding bubble.

(30) $$\frac{\partial C_{c}}{\partial t}+\frac{\varepsilon R^{2}\dot{R}}{r^{2}}\frac{\partial C_{c}}{\partial r}=a_{l}\left(\frac{\partial^{2}C_{c}}{\partial r^{2}}+\frac{2}{r}\frac{\partial C_{c}}{\partial r}\right)+\frac{\gamma D_{T}}{r}\frac{\partial C_{c}}{\partial r}$$

where $C_{c}$ is the concentration distribution, $a_{l}$ is the thermal diffusivity. $\varepsilon =1-\frac{\rho_{g}}{\rho_{l}}$ represents the dimensionless gas–liquid density ratio. γ is a negative value, given without loss of generality, the value γ=-1 for suction, and γ=0 for zero suction.

The final term, which arises from the suction process and produces a diffusion flux that is multiple in magnitude and directed in the opposite direction from the original one, represents the suction process on the concentration change. It receives its radial component from [[118], [119], [120]]:

(31) $$\vec{r}.\gamma a_{l}\nabla C_{c}=\frac{\gamma a_{l}}{r}\frac{\partial C_{c}}{\partial r}$$

The gas molar flux through the bubble surface is equal to the change rate of the gas molar concentration in the bubble [119,[121], [122], [123], [124], [125]]:

(32) $$\frac{1}{RT}\frac{d}{\mathrm{dt}}\left(\frac{4}{3}\pi R^{3}P_{g}\right)=4\pi R^{2}a_{l}{\left(\;\frac{\partial C_{c}}{\partial r}\right)}_{r=R}$$

The Laplace form depicts the pressure balance on the gas–liquid interface and takes into consideration the surface tension impact at the gas–liquid interface and the tissue viscoelastic impacts, is defined as [24,126,127]:

(33) $$P_{g}=P_{\mathrm{amb}}+\frac{2\sigma }{R}+\Omega \left(R\right)$$

In this instance, the pressure that the surrounding tissue exerts due to its deformation is represented by Ω(R). Gas bubbles develop in the biotissue when there is a sudden drop in atmospheric pressure, as happens when a diver quickly ascends from a dive or when a person flies long distances. Torrential pain in the joints and chest, skin rashes, cramps, and paralysis are symptoms of this phenomenon. An arterial gas embolism can result from expanding gas-induced pulmonary barotraumas, or sprains and ruptures in alveolar capillaries, which allow alveolar gas to enter the arterial circulation [65,116,128]. The gas typically migrates to tiny arteries with an average diameter of 30 to 60 μm when cerebral arterial gas embolization occurs. Fig. 6 illustrates two ways in which the emboli cause pathologic changes: a reduction in perfusion distal to the obstruction and an inflammatory response to the microbubble [116].

The results obtained of the decompression illness based on the dynamics of gas bubbles in the biotissue for different concentration models are summarized in Table 5, to illustrate the theoretical models and their solutions in the development of the decompression illness.

**Table 5:** Results of concentration models and behaviour of cavitation in decompression illness.

Diffusion equation	Solution	Cavitation radius	Model
$\frac{\partial C_{c}}{\partial t}+\frac{\varepsilon R^{2}\dot{R}}{r^{2}}\frac{\partial C_{c}}{\partial r}=a_{l}\left(\frac{\partial^{2}C_{c}}{\partial r^{2}}+\frac{2}{r}\frac{\partial C_{c}}{\partial r}\right)+\frac{\gamma D_{T}}{r}\frac{\partial C_{c}}{\partial r}$	$C_{c}\left(r,t\right)=C_{\infty }-k\left(t\right)\left(\left(1+\lambda a_{l}\left(\varepsilon +\frac{1}{2}\right)\right)\left(ln\left(\frac{R}{r}\right)\right)-ln\left(\frac{R}{R_{m}}\right)\right)+\lambda a_{l}\left(r-R_{m}\right)\left(\frac{r+R_{m}}{4R^{2}}+\frac{\varepsilon R}{rR_{m}}\right)$	$R\left(t\right)=\sqrt{\begin{array}{c} 2a_{l}\frac{-\frac{1}{3}ln\varphi_{0}-\frac{\Delta C_{0}}{k_{0}}}{\frac{1}{3}\left(\varepsilon +\frac{1}{2}\right)ln\varphi_{0}+\frac{1}{4}\varphi_{0}^{-\frac{2}{3}}+\varepsilon \left(1-\varphi_{0}^{\frac{1}{3}}\right)}\\ \left(t-t_{0}\right)+R_{0}^{2} \end{array}}$	Results in ref. [14]
$\frac{\partial C_{c}}{\partial t}=a_{l}\left(\frac{\partial^{2}C_{c}}{\partial r^{2}}+\frac{2}{r}\frac{\partial C_{c}}{\partial r}\right)$	$C_{c}\left(r,t\right)=C_{\infty }-A\left(exp\left(\frac{-\mu a_{l}}{2}\right)-\frac{R}{R_{m}}exp\left(\left(\frac{-\mu a_{l}}{2}\right){\left(\frac{R_{m}}{R}\right)}^{2}\right)\right)+A\left(\sqrt{\frac{\pi \mu a_{l}}{2}}\left(erf\left(\sqrt{\frac{\mu a_{l}}{2}}\right)-erf\left(\sqrt{\frac{\pi \mu a_{l}}{2}}-\frac{R_{m}}{R}\right)\right)\right)$	$R\left(t\right)=\sqrt{\begin{array}{c} 2a_{l}\frac{\frac{\Delta T_{0}}{A_{0}}-\left(\varphi_{0}^{\frac{1}{3}}-1\right)}{\left(\varphi_{0}^{\frac{1}{3}}-1\right)}\\ \left(t-t_{0}\right)+R_{0}^{2} \end{array}}$	Results in ref. [108,109]
$\frac{\varepsilon R^{2}\dot{R}}{r^{2}}\frac{\partial C_{c}}{\partial r}=a_{l}\left(\frac{\partial^{2}C_{c}}{\partial r^{2}}+\frac{2}{r}\frac{\partial C_{c}}{\partial r}\right)$	$C_{c}\left(r\right)=\frac{\begin{array}{c} e^{-\frac{\varepsilon kR}{r}}\left(C_{\infty }e^{\frac{\varepsilon kR}{R_{m}}}\left(e^{\varepsilon k}-e^{\frac{\varepsilon kR}{r}}\right)\right)+\\ C_{R}e^{\varepsilon k}\left(e^{\frac{\varepsilon kR}{r}}-e^{\frac{\varepsilon kR}{R_{m}}}\right) \end{array}}{e^{-\varepsilon k}-e^{-\varepsilon kR/R_{m}}}$	$R\left(t\right)=\sqrt{R_{0}^{2}+2R_{0}{\dot{R}}_{0}\left(t-t_{0}\right)}$	Results in ref. [119]

The modeling of gas bubble dynamics during decompression provides critical insight into the pathophysiology DCI. By capturing the expansion, migration, and collapse of inert gas bubbles in soft tissues, our model supports the identification of key mechanical thresholds that lead to tissue injury, vascular blockage, or inflammatory responses. These insights have direct implications for the design of safer decompression profiles in diving medicine, particularly in predicting risk zones where supersaturation and rapid gas diffusion may lead to harmful bubble nucleation. Furthermore, the model can aid in evaluating the efficacy of therapeutic interventions such as hyperbaric oxygen therapy, by estimating how pressure gradients influence bubble dissolution over time. Ultimately, integrating such dynamic modeling into decompression algorithms could contribute to more personalized and physiology-based dive planning, reducing the incidence and severity of DCI in at-risk populations such as divers, aviators, and astronauts.

## 7 Conclusions and outlook

In recent years, extensive research has been devoted to understanding the behavior and applications of microcavitation bubbles in therapeutic and biological systems, including histotripsy, cellular membrane disruption, bubble growth in biotissues of divers, and laser-induced cavitation in lithotripsy. Despite these efforts, the complex mechanical behavior of microcavitation bubbles, especially in viscoelastic and heterogeneous biological environments, still presents significant theoretical and practical challenges that require further investigation. This review focused on the fundamental and advanced aspects of microcavitation dynamics, including bubble growth in fluids and gel-like media, their nonlinear interaction with surrounding tissues, and their mechanical impact during various therapeutic procedures. We have systematically examined and compared various theoretical models such as Neo-Hookean elasticity and the Quadratic Law Kelvin-Voigt viscoelastic framework highlighting their assumptions, limitations, and applicability to real biological systems. Particular attention was given to their relevance in modeling lipid-coated bubbles, tissue ablation, and decompression-related pathology. Importantly, the theoretical frameworks reviewed here hold substantial translational value. In drug delivery, these models inform the control of microbubble rupture and localized therapeutic release. In histotripsy, they offer insight into the mechanical thresholds for tissue fractionation. In ultrasound imaging, they contribute to improving the stability and acoustic response of contrast agents. Furthermore, in decompression illness, the modeling of gas bubble evolution helps guide safer decompression protocols and therapeutic strategies. By bridging mathematical theory with biomedical relevance, this review aims to provide a unified perspective on microcavitation dynamics and their mechanical applications. Future research should continue to refine these models to incorporate more realistic tissue behaviors such as anisotropy, poroelasticity, and multiscale coupling. We hope that this work will assist researchers in resolving discrepancies between existing theoretical predictions and experimental findings, particularly in the context of therapeutic ultrasound and cancer cell treatment.

## Data availability statement

No new data were created or analyzed in this study.

## CRediT authorship contribution statement

**Ahmed K. Abu-Nab:** Writing – review & editing, Writing – original draft, Visualization, Validation, Supervision, Software, Resources, Methodology, Investigation, Formal analysis, Data curation, Conceptualization. **Adel M. Morad:** Formal analysis, Data curation, Conceptualization. **Ehab S. Selima:** Formal analysis, Data curation, Conceptualization. **Tetsuya Kanagawa:** Supervision, Funding acquisition, Formal analysis, Data curation, Conceptualization. **Ali F. Abu-Bakr:** Writing – original draft, Visualization, Supervision, Software, Resources, Formal analysis, Data curation, Conceptualization.

## Declaration of competing interest

The authors declare that they have no known competing financial interests or personal relationships that could have appeared to influence the work reported in this paper.
